# Evolutionary graph theory beyond pairwise interactions: Higher-order network motifs shape times to fixation in structured populations

**DOI:** 10.1371/journal.pcbi.1011905

**Published:** 2024-03-15

**Authors:** Yang Ping Kuo, Oana Carja

**Affiliations:** 1 Computational Biology Department, School of Computer Science, Carnegie Mellon University, Pittsburgh, Pennsylvania, United States of America; 2 Joint Carnegie Mellon University-University of Pittsburgh Ph.D. Program in Computational Biology, Carnegie Mellon University, Pittsburgh, Pennsylvania, United States of America; Max Planck Institute for Evolutionary Biology: Max-Planck-Institut fur Evolutionsbiologie, GERMANY

## Abstract

To design population topologies that can accelerate rates of solution discovery in directed evolution problems or for evolutionary optimization applications, we must first systematically understand how population structure shapes evolutionary outcome. Using the mathematical formalism of evolutionary graph theory, recent studies have shown how to topologically build networks of population interaction that increase probabilities of fixation of beneficial mutations, at the expense, however, of longer fixation times, which can slow down rates of evolution, under elevated mutation rate. Here we find that moving beyond dyadic interactions in population graphs is fundamental to explain the trade-offs between probabilities and times to fixation of new mutants in the population. We show that higher-order motifs, and in particular three-node structures, allow the tuning of times to fixation, without changes in probabilities of fixation. This gives a near-continuous control over achieving solutions that allow for a wide range of times to fixation. We apply our algorithms and analytic results to two evolutionary optimization problems and show that the rate of solution discovery can be tuned near continuously by adjusting the higher-order topology of the population. We show that the effects of population structure on the rate of evolution critically depend on the optimization landscape and find that decelerators, with longer times to fixation of new mutants, are able to reach the optimal solutions faster than accelerators in complex solution spaces. Our results highlight that no one population topology fits all optimization applications, and we provide analytic and computational tools that allow for the design of networks suitable for each specific task.

## Author summary

Accelerating the rate of solution discovery has been a long-standing goal in directed evolution and evolutionary optimization problems. One way to accelerate evolutionary search is to design population structures that control the mode and tempo of evolutionary dynamics. Population structure can amplify the action of selection and accelerate rates of evolution, or reversely, suppress selection and slow evolution down. Here we use evolutionary graph theory to systematically study how higher-order motifs in a population’s network of interaction and replacement can shape the velocity of evolution. In particular, we show that by changing the number of triangles (motifs of degree three) in a network, while fixing lower-order structures, we can continuously tune times to fixation of new variants in the population, independently of their probabilities of fixation. This allows us to design population structures that find optimal solutions across a broad spectrum of evolutionary optimization problems. We show that the best solutions need not always be the ones with the smallest times to fixation and discuss what motifs found in real biological networks can teach us about designing highly evolvable artificial ones.

## Introduction

The spatial structure of a population is a powerful determinant of a population’s evolutionary outcome. Some structures have topological properties that can speed up evolution and amplify the spread of a mutant with even the slightest selective advantage [1], while others work against the force of selection, increasing the role of evolutionary stochasticity and chance [2]. Evolutionary graph theory is the mathematical framework that formalizes the representation of complex population structure and its evolutionary effects [2–5]. In this framework, each individual occupies a node in a graph, and the edges represent the spatial or replacement patterns of interaction between neighboring nodes. The mode and tempo of evolution are studied through two main quantities: the probability of fixation of a new mutant in the population, which measures the likelihood that the mutant lineage takes over the entire population, and the mean time to fixation, the expected time until the population consists of only mutant descendants.

Most prior theoretical work has focused on studying probabilities of fixation, with theoretical explorations of times to fixation restricted to small networks [6], symmetric topologies such as lattices, rings, and stars [7–10] or sparse networks, where mutants grow in clusters [11, 12]. This imbalance of focus can be partially attributed to the assumption that the waiting time until a successful mutant appears in the population is much larger than the time it takes for this mutant to sweep through it. This would make the rate of evolution of the population mostly depend on the mutant’s probability of fixation. However, this assumption does not always hold, especially for engineering applications in directed evolution or evolutionary optimization problems with the goal of evolutionarily increasing rates of solution discovery [13–19]. These applications often utilize an elevated rate of mutation in order to speed up the generation of new candidate solutions and this accelerates search time by several orders of magnitude [20, 21]. In these contexts, fixation times of new mutants start to shape rates of evolution even more than probabilities of fixation, and selecting population structures purely for amplification or suppresion of selection could lead to a substantial evolutionary slowdown [11].

This has lead to recent interest in studying times to fixation, especially in the context of trade-offs with the probability of fixation [22, 23]. These studies have explored how to topologically build population graphs that increase probabilities of fixation of beneficial mutations, at the expense, however, of longer fixation times, which can slow down rates of evolution under elevated mutation rate. It remains an open question how to change a network’s topology in order to optimize times to fixation of new variants in the population, with negligible change to probabilities of fixation, i.e. to the amplification of selection.

**Fig 1:**
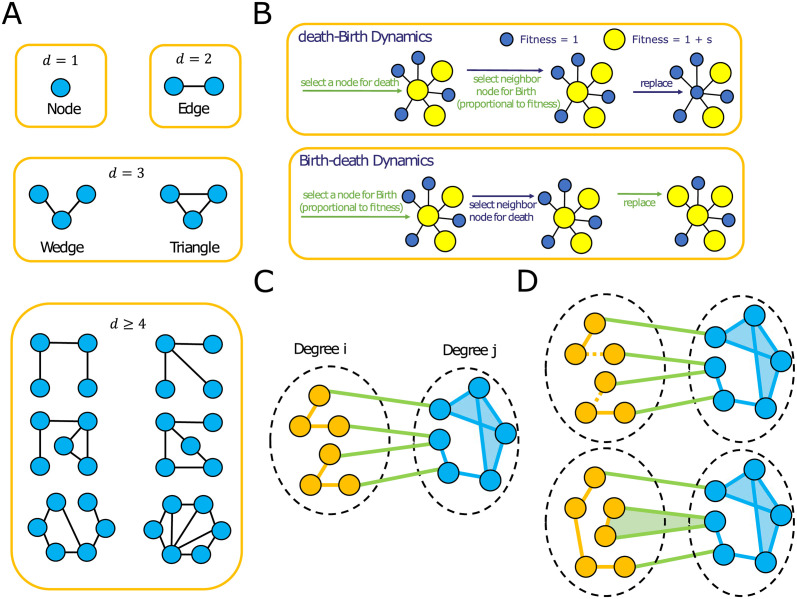
Illustration of the model. **Panel A** illustrates the levels of structural organization in the network. The list of motifs for *d* ≥ 4 is not exhaustive. **Panel B** illustrates the Bd (Birth-death) and the dB (death-Birth) update rules. **Panel C** shows the degree heterogeneous graphs we design, consisting of two groups of nodes with distinct degrees. **Panel D** illustrates the edge swap operation used to tune triangle fractions in the graph, without changing the degree distribution and mixing pattern of the network. Initially, there is no triangle consisting of mixed node degrees. We randomly select two edges of the same type (denoted by color) to be disconnected and nodes that were “parallel” with respect to the two disconnected edges are then connected, thus preserving the number of edges. After the rewiring step, there exists a triangle that connects two yellow nodes and a blue node. The degrees of the nodes and frequencies of edge type, however, are preserved.

While previous work has focused on the evolutionary role of pairwise node connections exclusively, many spatial patterns of replacement do not take place between pairs of nodes, but rather as collectives, at the level of groups of nodes [24, 25]. Networks exhibit higher-dimensional patterns of node interconnections, which can be organized by the number of nodes participating in forming the patterns (Fig 1A). Lower-order patterns of connectivity, that can be captured at the level of individual nodes (dimension *d* = 1) and edges (dimension *d* = 2), have been shown to significantly shape probabilities of fixation [5, 26–29] and are therefore unsuitable for shaping times to fixation, while keeping probabilities constant. Here we show that moving beyond dyadic structures is necessary to explain the trade-offs between probabilities and times to fixation of new mutations and to be able to understand the networks for which we can minimize or maximize times to fixation, without changing the probabilities of fixation.

We systematically explore how higher-dimensional network motifs (*d* ≥ 3), and, in particular, three-dimensional wedges and triangles, shape probabilities and times to fixation of a new mutant in the population and identify motifs that allow for continuous tuning of times to fixation, independent of fixation probabilities. We show that increasing the triangle count of a graph increases times to fixation, without influencing the probability of fixation. We also show that increasing the mean degree of the network not only decreases the time to fixation, but also diminishes the triangles’ ability to shape times to fixation. We find a weak increase with triangle count for the probability of fixation only for highly assortative graphs, but, since they are known suppressors of selection, this effect does not undermine the utility of tuning triangles counts for these networks [5]. This is because in applications where we want to reduce the rate of evolution, increasing time to fixation can achieve the same goal as decreasing the probability of fixation. Collectively, our results suggest that the fraction of triangle motifs in a graph is one of the most versatile parameters in controlling fixation acceleration.

We further show how to apply our algorithms and analytic results to two evolutionary optimization problems and show that the rate at which a population discovers the optimum can be tuned near continuously by adjusting the higher-order topology of the agent population. We highlight that the effects of population structure on the rate of solution discovery are more subtle than previously recognized, and find that decelerators with longer times to fixation are able to reach the optimal solution faster than accelerators when the space of possible solutions is rugged and complex. No one population structure is perfect for all optimization tasks, and careful consideration of the problem landscape is necessary for selecting the correct approach. Our work reinforces the importance of understanding and exploring common design principles of biological networks for engineering highly evolvable artificial systems.

## Model

We compute probabilities and times to fixation of a new mutation *a* that appears in an initially homogeneous population of *A*-type individuals and use a Moran birth-death process to track variant frequency changes. An individual with the *A* allele is assumed to have fitness one, while an individual with allele *a* has assigned fitness (1 + *s*). The population size is kept fixed at *N*.

We use unweighted, undirected graphs to represent the population structure. A node in the graph represents an individual in the population. The network edges are proxies for the local pattern of replacement and substitution: an edge can thus represent spatial proximity or the social architecture of interaction between individuals in the population. A node can also be interpreted as a homogeneous subgroup of individuals and the edges as migration corridors between them, with the assumption that the timescale of a mutation traveling between nodes is much larger than the time scale of fixation within a node [30–32].

We study both the Birth-death and the death-Birth process on these networks. These two update rules are equivalent in well-mixed populations, however they have been shown to lead to drastically different long-term evolutionary dynamics on graph structures [2, 5, 33]. In the Birth-death process, at every time step, a random individual is chosen for reproduction, proportional to fitness. An individual occupying a neighboring node is then randomly chosen for death and replaced by the new offspring. In the death-Birth update, an individual is first randomly chosen for death and is replaced by the offspring of a neighboring node. The neighbors of the vacant node compete for this vacancy and one neighbor is chosen for reproduction with probability proportional to fitness (Fig 1B).

Higher-order motifs create graph geometries produced by different patterns of interconnecting nodes. To describe and constrain random graphs by levels of organization in successively finer detail, [34] previously introduced the mathematical formalism of *dK*-distributions, where *d* denotes the dimension of an interaction involving *d* nodes. Thus, the 0*K*-distribution represents the average node degree. The 1*K*-distribution is the degree distribution of the graph, which specifies the fraction of nodes having degree *i* in a graph. 2*K* refers to the pattern of interactions, or mixing pattern, between two nodes. It is defined as the fraction of edges that connect a node of degree *i* to a node of degree *j*. Network assortativity, *r*, is also often used to describe mixing behavior in a network and is defined as the Pearson correlation coefficient of the degrees at either ends of an edge [35]. The 3*K* distribution describes interactions between groups of three nodes. For the 3*K* distribution there exist two possible connection topologies: wedges, chains of three nodes connected by two edges and their dimension three complement topology, triangles, cliques of three nodes (Fig 1A). Here we consider interactions of *d* ≥ 3 and particularly focus on the role of 3*K* interactions in shaping probabilities and times to fixation.

To systematically study the role of higher order motifs, the challenge lies in tuning these higher levels of organization while keeping lower levels constant. This is complicated by the fact that, for example, varying the number of triangles in the network also changes the degree distribution and the network mixing pattern, which we have previously shown to significantly affect probabilities of fixation [5].

We start by studying the role of higher order interactions in random regular graphs, where all the nodes have the same degree [36–38]. This simplifies the problem, since all *k*-regular graphs of fixed degree *k* share the same mixing pattern. We use degree-preserving edge swap operations to computationally tune the number of higher-order network motifs, while maintaining the lower level of organization, the degree distribution, constant [39]. We build algorithms that combine these approaches with simulated annealing to produce a sampling network generation algorithm.

For example, for tuning the transitivity or fraction of closed triangles *ϕ* over all triples in the network to a target fraction *ϕ*_*target*_, we accept an edge swap based on the Metropolis–Hastings criterion [40]

$$\begin{array}{c} \text{Uniform}\left[0,1\right]\leq \;\text{min}\;[1,\frac{P(\phi^{\prime }|\phi_{target})}{P(\phi |\phi_{target})}]. \end{array}$$ (1)

Here, *ϕ*′ is the fraction of triangles after the proposed edge-swap, and *P* is the probability density function of a Gaussian with mean *ϕ*_*target*_ and variance *σ*^2^. The algorithm always accepts edge swaps that bring the network closer to the target and accepts other edge swaps randomly. We increase *σ* by a factor of 1.001 every *Nk* times, where N is the size of the graph. We run the algorithm for *k*−million steps. Since edge swapping can cause the graph to become disconnected, we use the heuristic outlined in [41] to ensure graph connectivity. For regular graphs, the above algorithm will sample uniformly all connected regular graphs with the target fraction of triangles. This is because without the criterion in Eq (1), the Markov chain converges to a unique stationary distribution, which is the uniform distribution over the state space of all connected graphs with the same degree distribution [41]. The Markov chain with the condition in Eq (1) has a stationary distribution of $\frac{P(\phi |\phi_{target})}{\sum_{\phi^{\prime }}P\left(\phi^{\prime }|\phi_{target}\right)\left|G\left(k,\phi^{\prime }\right)\right|},$ where |*G*(*k*, *ϕ*′)| is the cardinality of connected graphs *G* with degree *k* and fraction of triangles *ϕ*′. In Section 3 in the S1 File, we also present an analysis of the *d* = 4 topology.

We expand this approach to degree-heterogeneous graphs by designing graphs with two distinct degrees (Fig 1C). This allows us to seamlessly tune the number of triangles without changing degree distributions and mixing patterns of the graphs. These graphs are designed starting with two random regular networks of size *n*_1_ and *n*_2_ and fixed degrees *k*_1_ and *k*_2_, and we use edge swaps to connect them. Here, the edge swap operation we previously used for the analysis of *k*-regular graphs is unsuitable, since that algorithm also alters the graph mixing pattern. This is not an issue for k-regular graphs, but not controlling for the mixing pattern of a graph with variance in degree can potentially lead to erroneous interpretations (see Fig A in the S1 File). For studying degree-heterogeneous networks, we instead use the *dK*-preserving rewiring, which is a generalization of the degree-preserving edge swap for higher-order motifs [34]. Fig 1D illustrates a 2*K*-preserving rewiring. Here, we replace the condition in Eq (1) with the following and alway accept the edge swap if the change in the fraction of triangles is towards the target, as well as according to

$$\begin{array}{c} \text{Uniform}\left[0,1\right]\leq \text{min}\;(1,\gamma ), \end{array}$$ (2)

if the change is in the other direction. Here, 1/*γ* is the annealing temperature which controls how stringent the criterion must be and is decreased as the graph generation algorithm proceeds. For degree-heterogeneous graphs, we are not guaranteed uniform sampling over the fraction of triangles.

Once the network structure is set, we use ensembles of at least 10, 000 Monte Carlo simulations, as well as analytic approaches, as described in the next section, to compute the probabilities and times to fixation of the new mutation in the population.

## Results

### The evolutionary role of higher order motifs in networks of uniform degree

We begin by studying the role of higher order network motifs in shaping rates of evolution for random regular graphs, where all nodes have the same degree *k*. Throughout this section any mention of fixation time refers to the conditional mean fixation time, or the average time to mutant fixation, conditional on fixation happening. We use the diffusion approximation [26, 42–44] to estimate the probability and time to fixation of the new *a* mutant as it appears in a random node of the network. Here, we describe the main steps and intuition behind the analytical approximation and provide the complete theoretical analysis for the Birth-death dynamics in Section 1 in the S1 File. We denote by *p*_*a*_ the mutant frequency in the population. There are three possible types of edges in the network: *AA*, *Aa*, *aa* and we denote their frequencies as *p*_*AA*_, *p*_*Aa*_, *p*_*aa*_. These frequencies can be expressed in terms of *p*_*a*_ and *p*_*Aa*_, with *p*_*AA*_ = (1 − *p*_*a*_) − *p*_*Aa*_ and *p*_*aa*_ = *p*_*a*_ − *p*_*Aa*_.

Since mutant frequency only changes when a replacement event occurs between edges connecting a wild-type and mutant, the whole dynamical system can be described by changes in *p*_*a*_ and *p*_*Aa*_. At every time point, *p*_*a*_ either remains the same, increases by 1/*N*, or decreases by 1/*N*. We can write the expected change μ in node frequency over one generation as

$$\begin{array}{c} \mu \left(\Delta p_{a}\right)=\frac{s}{w}p_{Aa}, \end{array}$$ (3)

where *w* is the mean fitness of the individuals in the population (see derivation in the S1 File).

Using pair approximations [45, 46], we compute the expected change in *Aa* edge-type frequency over one generation, *p*_*Aa*_, as

$$\begin{array}{c} \mu \left(\Delta p_{Aa}\right)=\frac{1}{k}p_{Aa}[\left(k-1\right)\left(1-\phi \right)(1-\frac{p_{Aa}}{p_{A}p_{a}})-1]+O\left(s\right). \end{array}$$ (4)

Observe that the expected change in mutant node frequency is of the order of the selection coefficient *s*, while the expected change in *Aa* edge frequency contains terms independent of *s*. This means that these two dynamical processes operate on different time scales and, in the limit of diminishing selection, the dynamics of the edges are faster than those of the nodes. We can therefore assume that the edge frequencies are at equilibrium in the timescale of the node dynamics. We can write *p*_*Aa*_ at equilibrium

$$\begin{array}{c} p_{Aa}^{*}=\left(1-F\right)p_{A}p_{a},\;\text{where}\;F=\frac{1}{\left(k-1)(1-\phi \right)}, \end{array}$$ (5)

which is the result of setting the first term in Eq (4) to zero and solving for *p*_*Aa*_. Using this approximation of *p*_*Aa*_, the Kolmogorov equation (Supplementary Equation (16)) reduces to depend on *p*_*a*_ alone. The probability of fixation of the *a* allele for any initial mutant frequency *P*(*p*_*a*_) is approximated by the solution to the equation:

$$\begin{array}{c} \frac{2+s}{2wN}\frac{\partial^{2}P}{\partial p_{a}^{2}}+\frac{s}{w}\frac{\partial P}{\partial p_{a}}=0. \end{array}$$ (6)

The approximate solution for this equation is $P\left(p_{a}\right)=\left(1-e^{-Nsp_{a}}\right)/\left(1-e^{-Ns}\right)$, for weak *s* [47]. This equation is identical to that corresponding to a well-mixed population, as expected for *k*-regular graphs [2]. The probability of fixation of the new mutant is therefore independent of the higher-order configuration of the nodes of the network.

While higher-order motifs may not shape probabilities of fixation, they can drastically change the time to fixation of the new variant. The time to fixation conditional on fixation, *T*(*p*_*a*_), can be found by solving

$$\begin{array}{c} \frac{2+s}{2wN}p_{Aa}\frac{\partial^{2}}{\partial p_{a}^{2}}\left[T\left(p_{a}\right)P\left(p_{a}\right)\right]+\frac{s}{w}p_{Aa}\frac{\partial }{\partial p_{a}}\left[T\left(p_{a}\right)P\left(p_{a}\right)\right]=-P\left(p_{a}\right), \end{array}$$ (7)

and the solution can be written as

$$\begin{array}{c} T\left(p_{a}\right)=\frac{1}{1-F}T_{wm}\left(p_{a}\right), \end{array}$$ (8)

where *T*_*wm*_(*p*_*a*_) is the conditional fixation time for the well-mixed model. We can obtain this value exactly using methods outlined in [47, 48].

**Fig 2:**
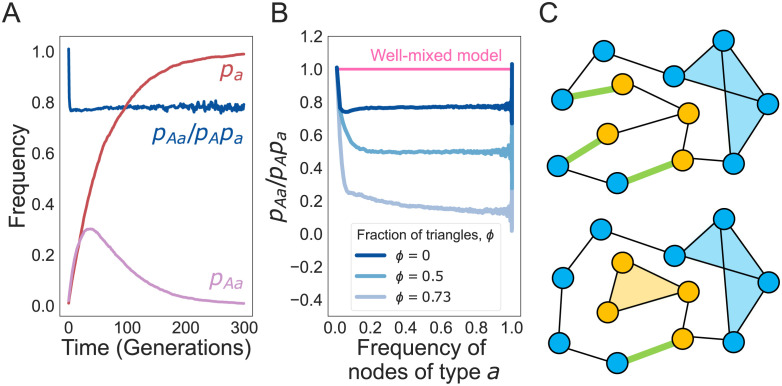
Triangles reduce the equilibrium number of *Aa* edges in the graph. **Panel A** shows the time trajectory of mean frequencies of *a* nodes (*p*_*a*_) and *Aa* edges (*p*_*Aa*_) using ensembles of Monte Carlo simulations on a 5-regular graph of size 100 with 0 triangles. Only trajectories that fix in *a* were used. **Panel B** shows the ratio between the frequency of *Aa* and the frequency of *a* nodes on graphs with various fractions of triangles *ϕ*. All results were obtained from 1000 replicate simulations. **Panel C** shows two graphs with the same degree distribution. The top graph has two triangles while the bottom one has three triangles. The number of open edges in green (edges that connect pairs of different genotype) is reduced when triangles are introduced.

The analytic approach outlined above provides intuition into why higher order motifs change times, but not probabilities of fixation for new mutants in the population. The equilibrium frequency of *Aa* edges is minimized in a population of all *A* or all *a* individuals (Fig 2A) and, as the frequency of *a* increases, the frequency of *Aa* edges quickly converges to a constant equal to (1 − *F*) times the product of the frequencies of *A* and *a*. This constant decreases with the fraction of triangles *ϕ* (Fig 2B) and this decrease leads to higher times to fixation for the new variants in the population. The parameter *F* in Eq (8) is analogous to measures of inbreeding due to population structure. Intuitively, rewiring a network of fixed degree distribution to introduce triangles (Fig 2C) increases the probability that two nodes with the same common ancestor are connected, thereby reducing the number of edges connecting nodes of different genotypes.

**Fig 3:**
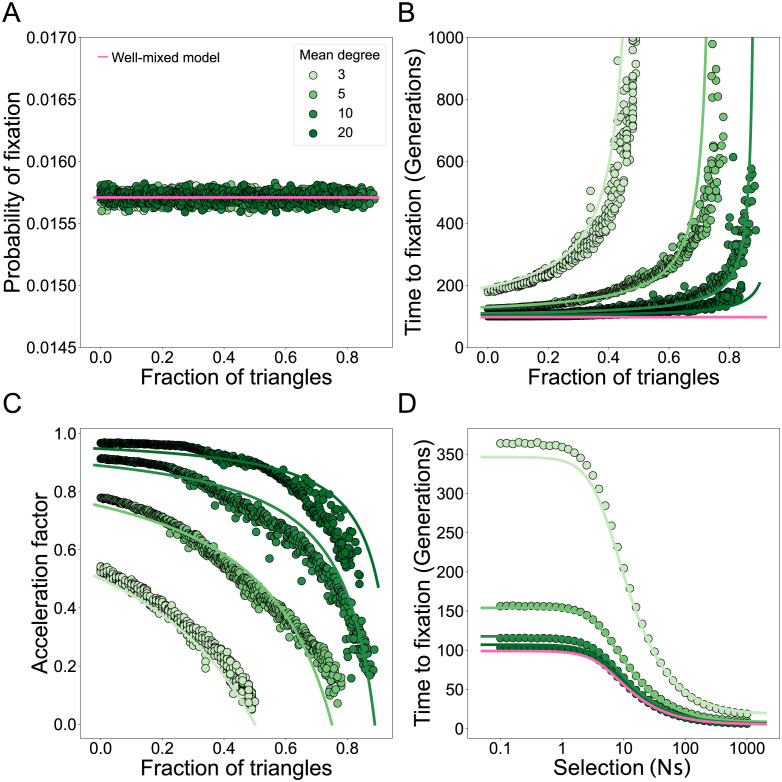
The fraction of triangles increases time to fixation in degree-uniform graphs. Each dot represents the ensemble average using 10^7^ replicate Monte Carlo simulations per graph, while the lines represent our analytical approximations. Here the degree distribution is held constant as we vary the fraction of triangles in the graphs, *N* = 100 and *s* = 0.01, such that *Ns* = 1. The color indicates the mean degree of the network, as in the legend. The fraction of triangles in the graph is tuned using edge swapping operations. **Panel A** shows the fixation probability. The line shows the solution to Eq (6). **Panel B** shows the fixation time. The lines show the solution from (8), with *F* from Eq (5). **Panel C** shows the acceleration factor as a function of the fraction of triangles. Lines show acceleration factor (1 − *F*), with *F* from Eq (5). **Panel D** shows the time to fixation as a function of selection strength. Lines show solutions to Eq (8), with *F* from Eq (5).

We show the accuracy of the analytic approximation for random regular networks in Fig 3. The fixation probability is independent of the node degree (Fig 3A) and, as the fraction of triangles *ϕ* increases, time to fixation increases, as *F* approaches 1 (Fig 3B). Analogous to the amplification parameter when studying probabilities of fixation [5], we define the acceleration factor of a graph as the time to fixation for an equivalent mutant in a well-mixed population divided by the time to fixation in a structured population. For *k*-regular graphs, the acceleration parameter is approximately (1 − *F*), which increases as the mean degree increases and the fraction of triangles decreases (Fig 3C). Our analysis highlights that *k*-regular graphs are not accelerators of fixation, since the acceleration parameter cannot exceed one. We also show that our approximation of time to fixation holds for a wide range of selection strengths (Fig 3D), even though in our derivation we assume weak selection.

### Higher-order network motifs increase time to fixation in degree-heterogenous networks

**Fig 4:**
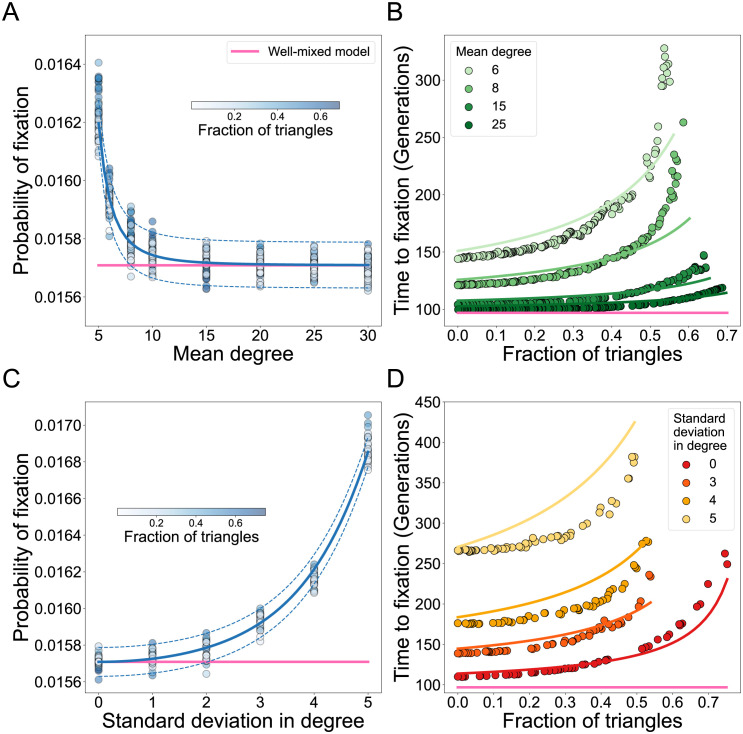
Higher-order interactions increase time to fixation in degree-heterogenous graphs with zero assortativity. Here the graph mixing pattern is constant, with assortativity equal to zero, *N* = 100 and *s* = 0.01, such that *Ns* = 1. **Panel A** shows the fixation probability as a function of the fraction of triangles and mean degree of the graph. Standard deviation in degree equal to two. **Panel B** shows the time to fixation, and the colors indicate the mean degree of the network as in the legend. Standard deviation in degree equal to two. **Panel C** shows the fixation probability as a function of the fraction of triangles and the heterogeneity in the degree of the graph. **Panel D** shows the time to fixation. For **Panels C** and **D**, networks of mean degree equal to eight are used. Each dot represents ensemble averages across 10^7^ replicate Monte Carlo simulations per graph, while the lines represent our analytic approximations. The probability of fixation is approximated using Supplementary Equations (41), (48) and (52), for graphs with 0 triangles. Confidence intervals according to Eq (10). The time to fixation is calculated using Supplementary Equations (41), (49) and (53).

To assess the evolutionary role of graph assortativity, we use the algorithm from [5] to design graphs with a fixed degree distribution and varying assortativity or mixing pattern. For degree-heterogeneous networks where the node assortativity is zero, the fraction of triangles similarly increases the time to fixation (Fig 4B and 4D), while having a negligible effect on the probability of fixation (Fig 4A and 4C). In these networks, we vary mean and standard deviation in degree independently and observe that the effects of triangles are more pronounced in networks of small mean degree and high standard deviation in degree. To show that the effect on the probability of fixation is negligible, we compare probabilities of fixation with the analytically approximated values for graphs without triangles:

$$\begin{array}{c} P\left(p_{a}=1/N\right)=\frac{1-e^{-\alpha s}}{1-e^{-N\alpha s}}, \end{array}$$ (9)

where *α* is the amplification factor calculated from Supplementary Equation (52). Since fixation is a Bernoulli random variable, we can also compute confidence intervals using

$$\begin{array}{c} \text{Var}\left[P_{MC}\right]=\frac{P(1/N)[1-P(1/N)]}{n_{MC}}, \end{array}$$ (10)

where *P*_*MC*_ is the estimate of fixation probability using *n*_*MC*_ Monte Carlo simulations. We show these approximations for graphs with zero triangles, along with confidence intervals, in Fig 4A and 4C. Graphs with a strictly positive number of triangles tend to be distributed around both sides of this approximation and are contained within two standard deviations of the approximated mean.

The analytic approximation for the fixation time on degree-heterogenous networks is presented in Section 2 in S1 File. The analytic approximation tends to slightly overestimate fixation time compared to stochastic simulations for graphs with high standard deviation in degree (Fig 4B and 4D). This is because our analytic approximation assumes that triangle motifs are distributed uniformly across all triplet types. However, the algorithm preferentially over-tunes the number of triangles using triples with nodes of high degree.

**Fig 5:**
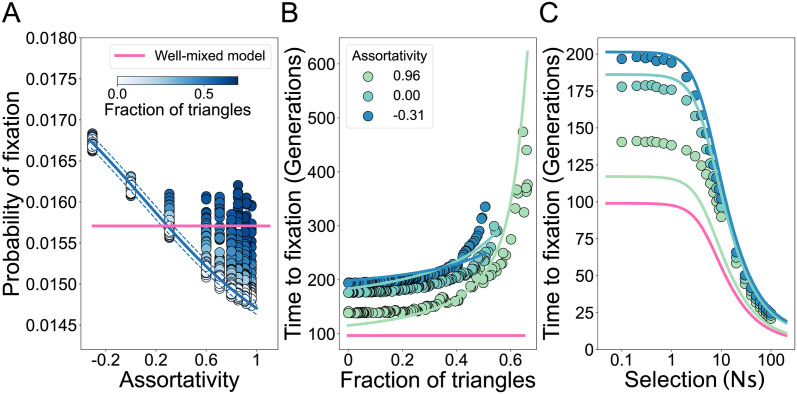
The probability and time to fixation in graphs with non-zero assortativity. The degree distribution of the network and its assortativity is held constant, as we vary the fraction of triangles in the graphs. Here, *N* = 100 and *s* = 0.01. The degree distribution is 50% nodes of degree 12 and 50% nodes of degree 4, such that the mean is 8 and the standard deviation is 4. **Panel A** shows the probability of fixation as a fraction of triangles and assortativity of the graph. **Panel B** shows the time to fixation and the colors indicate the assortativity of the network, as in the legend. **Panel C** shows the effect of the strength of selection for graphs with different assortativity and a fixed fraction of triangles equal to zero. Each dot represents ensemble averages using 10^7^ replicate Monte Carlo simulations per graph, while the lines are our analytic approximations. The probability of fixation is approximated using Supplementary Equations (41), (48) and (52), for graphs with 0 triangles. Confidence intervals according to Eq (10). The time to fixation is calculated using Supplementary Equations (41), (49) and (53).

For positive network assortativity, the frequency of higher-order motifs starts to change both the probability and the time to fixation of the new mutant (Fig 5). Fig 5A shows that the fraction of triangles has negligible influence on fixation probability for disassortative and weakly assortative graphs. However, as we increase assortativity, the probability of fixation increases for networks with many triangles. This observation can be explained by considering the expected number of *Aa* edges in the network. Similar to the case of *k*-regular networks, triangles reduce the number of *Aa* edges in the network (Fig B in S1 File). The key difference here is that regular graphs only have one type of *Aa* edges, while more heterogeneous networks have multiple types of *Aa* edges, as defined by the various degrees of the nodes they connect, *A*_*i*_*a*_*j*_. If the *A*_*i*_*a*_*j*_ edges are reduced by the fraction of network triangles by roughly the same amount, their effects cancel out and the new mutant’s probability of fixation remains unchanged. For highly assortative graphs however, *A*_*i*_*a*_*j*_ edge frequencies tend to decrease more for edges that connect nodes of low degree. The same behavior is observed across a wide range of graph families (see Figs C, D and E in S1 File). The increase in the time to fixation due to graph triangles is amplified by network assortativity (Fig 5B). In Fig 5C we show that the deceleration in fixation for networks with a fixed fraction of triangles is constant across biologically relevant selection ranges *Ns*, from 0.1 to 100. This deceleration is maintained across a wide range of selection strengths, suggesting that these networks are stable decelerators.

**Fig 6:**
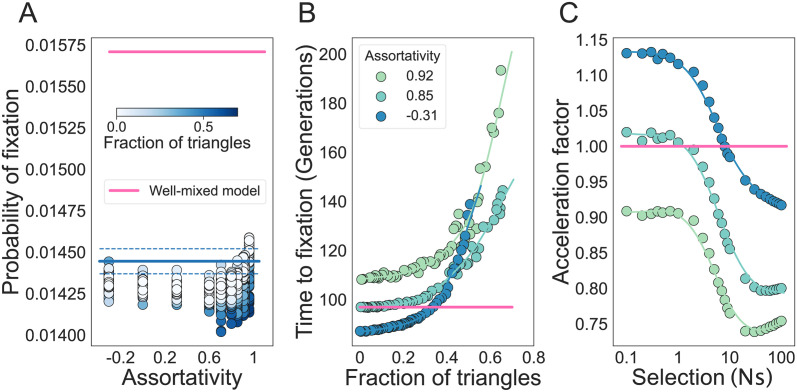
Population structures that accelerate the time to fixation are abundant, but only under death-Birth dynamics. **Panel A** shows the probability of fixation with respect to assortativity and fraction of triangles of the graph. **Panel B** shows the time to fixation and the colors indicate the assortativity of the network, as in the legend. **Panel C** shows the effect of the strength of selection for graphs with different assortativity and a fixed fraction of triangles equal to zero. Here the degree distribution of the network and its assortativity is held constant, as we change the fraction of triangles in the graphs. *N* = 100 and *s* = 0.01. The degree distribution is 50% nodes of degree 12 and 50% nodes of degree 4, such that the mean is 8 and the standard deviation is 4. Each dot represents ensemble averages using 10^7^ replicate Monte Carlo simulations per graph, while the lines are our analytic approximations. The probability of fixation is approximated for networks with zero triangles from Eq (9) with amplification factor equal to ${\left(E\left[i\right]\right)}^{2}/E\left[i^{2}\right]$, where the expectation is with respect to *i*, the degree of the network [27]. The lines for times to fixation and the acceleration factors are given by cubic spline regressions.

The role of triangles is invariant to the update process: under a death-Birth process, the fraction of triangles has minimal effects on the probability of fixation (Fig 6A) and the time to fixation similarly increases with an increased fraction of triangles (Fig 6B). However, we find that graphs with low assortativity and a low fraction of triangles exhibit a faster time to fixation compared to the well-mixed model. This acceleration, however, is not stable with respect to the strength of selection. We introduce the term piece-wise accelerators to describe these graphs, since they accelerate fixation for some fitness regimes, but decelerate it for others. Fig 6C shows that the acceleration factor decreases as the strength of selection increases, turning accelerators into decelerators. While all graphs under the Birth-death process are decelerators of fixation, we find that a piece-wise accelerator of fixation is prevalent under the death-Birth update rule.

### Optimizing network structure for evolutionary algorithm applications

**Fig 7:**
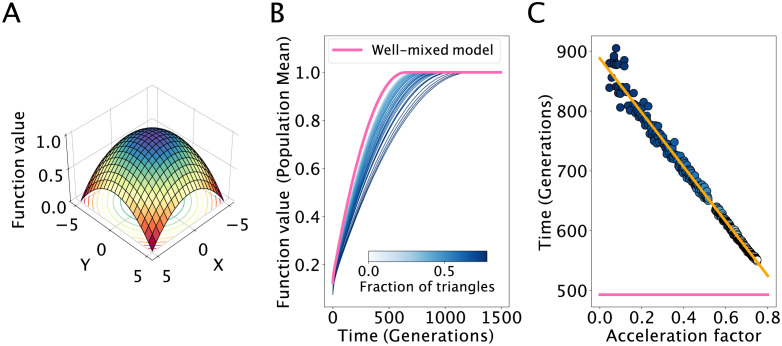
Effects of network topology on rates of optimization for smooth landscapes. We use population structures with mean degree five and varying *ϕ*. The results are averaged from 500 replicate simulations. **Panel A** illustrates the quadratic function, used as test function for optimization. In **Panel B** the lines represent average trajectories of the mean population function value. In **Panel C** the dots represent the average time to reach mean population function value of 0.95. Here the acceleration factor is (1 − *F*) calculated using Eq (5). The orange line represents linear regression, with *r* = −0.996.

Our results can be used to design networks with tunable probabilities and times to fixation. We first explore the role of network motifs in evolving a population to reach an optimal value on a smooth quadratic surface. We use a variation of the evolutionary algorithm on graphs described in [49]. Individuals in the population are represented by a two-dimensional vector which can be interpreted as the genotype of this individual and is mapped to a functional value (Fig 7A). The goal is to maximize the mean function value over the population. The fitness of the individual is calculated by its rank in the population, where the individual with the highest function value is assigned fitness of (1 + *s*), decreasing linearly to (1 − *s*). Such a selection scheme is called rank selection and maintains constant selection pressure on the population, independent of the problem [50]. The population evolves under the Birth-death process. For the example presented, we use 5-regular graphs of size *N* = 100 and a selection strength of *s* = 1, such that *Ns* = 100. Every time a node reproduces, either the parent or the offspring node mutates by adding a Gaussian noise vector to the genotype vector. The Gaussian noise has a mean of zero and standard deviation of 0.012.

Increasing the fraction of triangles in the network reduces the rate that the population discovers the optimal agent (Fig 7B). The population structure with the highest number of triangles has the lowest rate of convergence to an optimal reward of one, and the rate of convergence increases as the fraction of triangles decreases. Fig 7C shows the time it takes for each topology to reach a function value of 0.95. As expected, the time to reach the target increases as a function of the fraction of triangles in the network and decreases as a function of the network acceleration factor.

**Fig 8:**
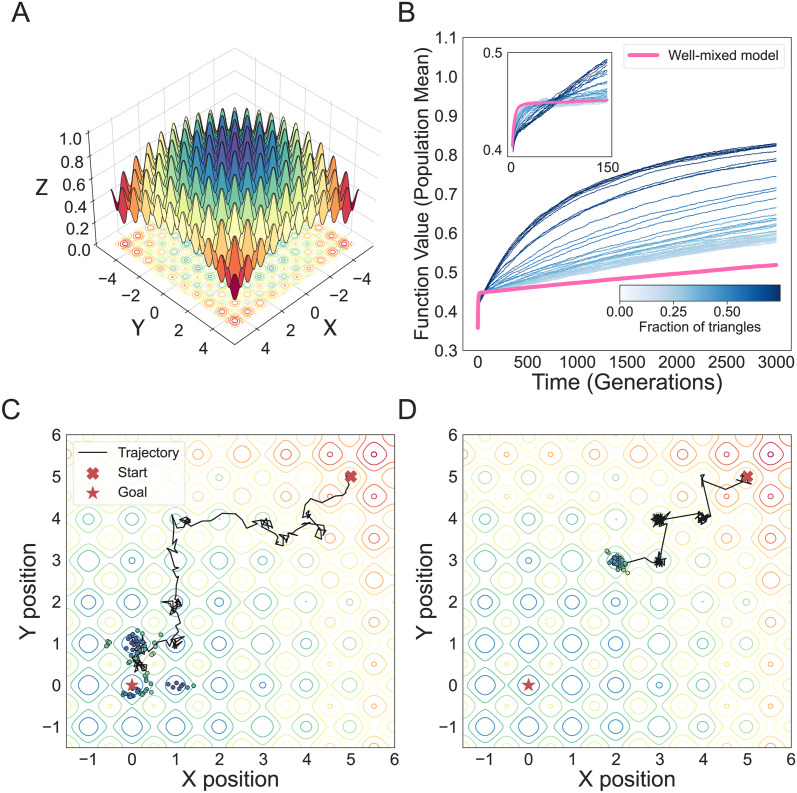
Advantage of increased fixation time in complex optimization problems. The population structures used in the simulation have mean degree of five and varying transitivity *ϕ*. The results are averaged from 1000 replicate simulations per graph. **Panel A** illustrates the Rastrigin function, used as test function. **Panel B** shows mean solution trajectories. **Panel C** shows the population trajectory and the distribution of solutions in function space for the network population, while **Panel D** shows the population trajectory the distribution of solutions in function space for a well-mixed population.

While triangles in a network may be detrimental in optimization problems on smooth fitness landscapes, in problems where the optimization landscape is rugged, increasing the fraction of triangles can lead to a population with an increased rate of solution discovery. To showcase this, the second application we consider is finding the global minimum of the Rastrigin function [51]. We invert the function, turning the problem into a maximization problem. The Rastrigin function has tunable ruggedness, which makes the gradient-based optimization methods used in convex optimization unsuitable (Fig 8A). Each individual in the population is represented by a vector in the domain of the Rastrigin function and fitness is mapped to the population ranking of the function value corresponding to the individual. The population reproduces on networks under the Birth-death process and Gaussian mutations occur randomly to change the offspring or the parent node. The Gaussian noise has a mean of zero and we use a standard deviation of 0.12, 10 times higher than the quadratic function case, since this problem is computationally much slower. We show that the population structure with the lowest number of triangles has a higher rate of initial convergence (Fig 8B), but it is soon overtaken by populations with higher numbers of triangles in the network (Fig 8C and 8D). This is because, for these types of problems, the best performers are population structures that increase time to solution discovery by allowing for more exploration on the rugged landscape.

## Discussion

To design spatial structures with accelerated (or decelerated) rates of evolution, we must first build a systematic understanding of how the spatial arrangement of a population shapes probabilities and times to fixation of new mutants entering a population. Of general interest are graph structures that can greatly amplify selection, but pay minimal cost in the time it takes for the mutant to sweep through and fix in the population. This type of structure is theorized to be desirable for two reasons: 1) fixation probability is important when the rate-limiting step is waiting for an advantageous mutant to occur; 2) fixation time is important in high mutation regimes, where the abundance of beneficial mutations negates loss due to stochastic drift. There are various approaches to finding such structures. For example, [22] developed a genetic algorithm that explores a space of connected graphs to optimize for fixation probability and fixation time. Separately, “*α*-bipartite” graphs [23] and “selection reactors” [52] use weighted edges to push the boundary of tradeoff between fixation probability and time.

Here, we take a different approach by focusing on undirected and unweighted graphs and build a systematic understanding of how the different levels of structural organization of a network shape evolutionary dynamics. Our results show that 3-dimensional structural motifs are the most effective network property for tuning temporal dynamics and times to fixation of new mutants in the population. While our most novel finding involves triangles and higher-order network motifs, our analysis also reveals connections between the evolutionary role of lower-levels of network organization (the node and edges) and previous population structure models. This is because the heterogeneous graph families we explore can, in some cases, be topologically mapped to classical deme-based population structures. For example, groups of nodes with distinct degrees can be thought of as population subdivisions, and the edges connecting these groups can be viewed as migration paths between demes. When the node degrees of the groups are identical (as in the case of k-regular graphs), we find that higher order networks motifs do not change probabilities of fixation, only times to fixation and this behavior qualitatively mirrors what has been observed in previous classical models [53, 54]. More generally, we find that the number of closed triad interactions influences fixation time with minimal impact on fixation probabilities when the graph is not assortatively mixed. Triad interactions alter evolutionary outcome similar to the migration rate in a deme model, although through a completely different mechanism.

The key distinction between the network-based models and previous deme-based models lies in the fact that deme models do not consider the structural properties within the subdivision, beyond the deme size. In contrast, the Moran process on graphs inherently models properties such as the number of connections, triad interactions, and higher-order connections within node groups. When there are differences in these structural properties between node groups, in addition to the quantitative differences observed in fixation time, we also observe qualitative deviations in fixation probabilities. Structural properties like degree distribution and mixing patterns tend to simultaneously affect fixation probability and fixation time, as these two quantities are closely intertwined [5, 23]. Here we find that the number of triangle motifs can affect both probabilities and times to fixation for graphs with non-zero assortativity.

While the field of evolutionary graph theory is usually interested in accelerators that minimize time to fixation, we also use tuned network structures for two very different optimization problems and show that accelerators don’t necessarily lead to faster rates of adaptation. Optimal structures depend heavily on the fitness landscape considered, as predicted by Sewell Wright’s shifting balance theory [55]. One case where faster time to fixation can hurt the long term adaptation of a population is when the fitness landscape consists of multiple fitness peaks separated by valleys. Here, an accelerator can prevent individuals from adequately exploring these valleys to eventually discover higher fitness peaks. We find that graphs with reduced fraction of triangles do well when evolving populations to solve optimization problems on smooth landscape, however, the same graphs can actually lead to slower solution discovery when the optimization landscape is produced from a rugged Rastrigin function. The observed fitness trajectory shows similar tortoise-and-hare pattern seen in structured bacterial population [56], indicating that our observation is the consequence of the shifting balance process. Our results highlight the need to search for structures outside of fast amplifiers, as the diversity of evolution and optimization landscapes dictates that certain structural properties are preferred in specific contexts, but no single set of properties is ideal for all scenarios. Moreover, we’ve demonstrated that degree distribution, mixing patterns, and triangle counts are effective network properties for systematically exploring the space of potential evolutionary outcomes within structured populations.

Our theoretical treatment is limited to studying probabilities and times to fixation, but more factors contribute to both natural and artificial evolving systems. For example, evolutionary algorithms often use high mutation rates and multiple mutants are available simultaneously in a population. Different clones compete and can interfere with the fixation of one another [57]. Effective traversal of the fitness landscape also relies on the existence and maintenance of mutation islands. Fixation time might not be the most direct quantity that describes the clonal dynamics in such a population and tools that study clonal interference could potentially also provide additional insights into the problem. Our work nonetheless provides the first exploration of the role of higher-order network motifs in shaping evolutionary dynamics and highlights the importance of using evolutionary design principles to engineer highly evolvable, adaptable artificial systems.

## Supporting information

S1 File The Supplementary material contains the complete analytical derivations and the supplementary figures associated with the manuscript. (PDF)
