# Supplementary material for: Evolutionary graph theory beyond pairwise interactions: Higher-order network motifs shape times to fixation in structured populations
Source: PLoS Comput Biol. 2024 Mar 15;20(3):e1011905. doi: 10.1371/journal.pcbi.1011905 (PMC10971782; doi:10.1371/journal.pcbi.1011905)
Supplement: S1 File — (PDF) [file pcbi.1011905.s001.pdf]

Supplementary Material: Evolutionary graph theory beyond pairwise interactions: higher-order network motifs shape times to fixation in structured populations

Yang Ping Kuo[1,2], Oana Carja[1,*],

1 Computational Biology Department, School of Computer Science, Carnegie Mellon University, Pittsburgh, Pennsylvania, USA

2 Joint Carnegie Mellon University-University of Pittsburgh Ph.D. Program in Computational Biology, Carnegie Mellon University, Pittsburgh, Pennsylvania, USA

* oana@cmu.edu

Contents

1 Analytic approximation for k-regular graphs 1

2 Analytic approximation for graphs of heterogeneous degree 7

3 Effects of 4-node Motifs on Evolutionary Dynamics 16

1 Analytic approximation for k-regular graphs

We start by presenting the full description of our analytic approach for k-regular graphs under the Birth-death update rule. A population of size N consisting of only A individuals is distributed over the nodes of a k-regular graph (each node has k neighbors). Using the diffusion approximation, we compute the probability and time to fixation, conditional on fixation, of a new mutant a appearing in a random node on the graph. We adopt an approach similar to [1] and use pair approximations to write higher-order interaction dynamics as approximations of lower-order ones.

At every time step, let p_a and p_A denote the frequencies of the mutant a and wild-type A in the population. Let p_{aa}, p_{aA}, p_{Aa} and p_{AA} denote the frequencies of the edge types and let $p_{Y|X}$ denote the probability of

observing an XY edge given the first node is type X. Here, X and Y can be A or a. Note that $p_{Aa} = p_{aA}$, $p_{aA} = p_{a|A} p_A$ and $p_{A|X} + p_{a|X} = 1$.

Let $P\left(\Delta p_a = +\frac{1}{N}\right)$ and $P\left(\Delta p_a = -\frac{1}{N}\right)$ denote the probabilities that an individual changes its allelic type towards or away from the mutant type a. Since mutant frequency only changes when a replacement event occurs between edges connecting a wild-type and mutant, the whole dynamical system can be described by changes in p_a and p_{Aa}.

The mutant frequency increases by $1/N$ in the population when a mutant a is selected for reproduction and a neighbor A is selected to be replaced. Therefore, p_a increases by $1/N$ with probability

$$\begin{aligned} P\left(\Delta p_a = +\frac{1}{N}\right) &= \left(\frac{1+s}{w} p_a\right) \sum_{k_A}^{k} \text{Prob}(k_A|a) \frac{k_A}{k} \\ &= \left(\frac{1+s}{w} p_a\right) \frac{1}{k} \mathbb{E}_{k_A}[k_A|a], \end{aligned} \tag{1}$$

where k_A and k_a denote the number of A and a neighbors of the focal mutant node and w represents the mean fitness of the population. We have $k_A + k_a = k$. The term $(1+s)p_a/w$ corresponds to the probability of selecting a mutant a for reproduction. The rest of the term corresponds to the probability of selecting a wild-type A to be replaced.

In pair approximations, the conditional probability $p_{A|a}$ that a neighbor is a wild-type A given that the reproducing node is a mutant a, is assumed to be independent of the other neighbors [2]. Then, the probability $\text{Prob}(k_A|a)$ that a has k_A neighbors, is the binomial distribution with k trials and probability $p_{A|a}$. The conditional expected number of A neighbors is therefore $kp_{A|a}$. This simplifies equation (1) to

$$\begin{aligned} P\left(\Delta p_a = +\frac{1}{N}\right) &= \frac{1+s}{w} p_a \frac{1}{k} k p_{A|a} \\ &= \frac{1+s}{w} p_{Aa}. \end{aligned} \tag{2}$$

Similarly, we can write the probability that the mutant frequency decreases by $1/N$,

$$P\left(\Delta p_a = -\frac{1}{N}\right) = \frac{1}{w} p_{Aa}. \tag{3}$$

Therefore, the first moment of the change in frequency of the mutant allele a, at every time step, can be

written as:

$$\begin{aligned}\mathbb{E}[\Delta p_a] &= \sum_{\Delta p_a} P\left(\Delta p_a\right)\Delta p_a \\ &= \frac{1}{N}\left(\frac{1+s}{w}p_{Aa} - \frac{1}{w}p_{Aa}\right) \\ &= \frac{s}{Nw}p_{Aa}.\end{aligned} \tag{4}$$

The second moment of the change in frequency of the mutant allele a, at every time step, can be written as:

$$\begin{aligned}\mathbb{E}[(\Delta p_a)^2] &= \sum_{\Delta p_a} P\left(\Delta p_a\right)\left(\Delta p_a\right)^2 \\ &= \frac{1}{N^2}\left(\frac{1+s}{w}p_{Aa} + \frac{1}{w}p_{Aa}\right) \\ &= \frac{2+s}{N^2 w}p_{Aa}.\end{aligned} \tag{5}$$

The variance of the change in frequency of the mutant allele a, at every time step, can be written as:

$$\begin{aligned}\mathrm{Var}[\Delta p_a] &= \mathbb{E}[(\Delta p_a)^2] - \mathbb{E}[\Delta p_a]^2 \\ &= \frac{2+s}{N^2 w}p_{Aa} - \frac{s^2}{Nw^2}p_{Aa}^2.\end{aligned} \tag{6}$$

The expectation and variance of the change in frequency of the mutant allele a, per generation (one generation consisting of N steps), can be written as:

$$\mu(\Delta p_a) = \frac{\mathbb{E}[\Delta p_a]}{\Delta t} = N\mathbb{E}[\Delta p_a] = \frac{s}{w}p_{Aa}, \quad \sigma(\Delta p_a) = \frac{\mathbb{E}[(\Delta p_a)^2]}{\Delta t} \approx \frac{2+s}{Nw}p_{Aa}, \tag{7}$$

in the limit of weak selection.

We next compute the first moment of the change in frequency of the edges of type Aa, p_{Aa}. The number of Aa edges only changes when the mutant a replaces a wild-type A and vice versa.

Firstly, when a replaces an A, the number of Aa edges decreases by one, but that is not the only edge type-changing. We also need to consider edges connecting the death node A to nodes other than the birth node a. Let us denote these nodes a' or A'. The AA' edges connecting the node to be replaced A to neighboring A' nodes become aA' edges and all Aa' edges connecting neighboring a' nodes to the death node A become aa' edges. This is represented by aAA' becoming aaA' and aAa' becoming aaa' in terms of triplet configuration. Let $k_{a'}$ be the number of aAa' configurations, and $k_{A'}$ be the number of aaA' configurations. Here, $k_{a'} + k_{A'} = k - 1$. In total, we lose $(k_{a'} + 1)$ Aa edges (the aA edge plus the $k_{a'}$ aA edges) and gain $k_{A'}$ aA edges. The probability of this event is the product of the probability of an a node replacing an A

node and the probability of the dead A node having exactly $k_{a'}$ a' neighbors and $k_{A'}$ A' neighbors. The probability that the frequency of Aa edges increases by $\frac{k_{A'}-k_{a'}-1}{2Nk}$ can be written as:

$$\begin{aligned} P\Big(\Delta p_{Aa} = \frac{k_{A'} - k_{a'} - 1}{2Nk}\Big) &= P\Big(\Delta p_a = +\frac{1}{N}\Big)\text{Prob}(k_{A'}|aA) \\ &= \frac{1+s}{w} p_{Aa}\text{Prob}(k_A|aA). \end{aligned} \tag{8}$$

Similarly, we can write the equivalent probability, for the event of a wild-type node A replacing a mutant node a, i.e. AaA' becoming AAA' and Aaa' becoming AAa':

$$\begin{aligned} P\Big(\Delta p_{Aa} = \frac{k_{a'} - k_{A'} - 1}{2Nk}\Big) &= P\Big(\Delta p_a = -\frac{1}{N}\Big)\text{Prob}(k_{A'}|Aa) \\ &= \frac{1}{w} p_{Aa}\text{Prob}(k_{A'}|Aa). \end{aligned} \tag{9}$$

The expected change in frequency of the Aa-type edges is sum of the expected change under these two possible replacement events. The expected change under the first event (a replaces A) is

$$\begin{aligned} \sum_{k_{A'}} \frac{k_{A'} - k_{a'} - 1}{2Nk} P\Big(\Delta p_{Aa} = \frac{k_{A'} - k_{a'} - 1}{2Nk}\Big) &= \frac{1+s}{w} p_{Aa} \sum_{k_{A'}} \frac{k_{A'} - k_{a'} - 1}{2Nk}\text{Prob}(k_{A'}|aA) \\ &= \frac{1+s}{w} p_{Aa} \sum_{k_{A'}} \frac{2k_{A'} - k}{2Nk}\text{Prob}(k_{A'}|aA). \\ &= \frac{1+s}{w}\frac{1}{2Nk} p_{Aa}(2\mathbb{E}[k'_A|aA] - k). \\ &= \frac{1+s}{w}\frac{1}{2Nk} p_{Aa}(2(k-1)p_{A|aA} - k). \end{aligned} \tag{10}$$

Here, we use the pair approximation again, and assume the probability of observing a wild-type A' as a neighbor of the replaced node is independent of other neighbors beside the reproducing node. Then, the probability of observing $k_{A'}$ aAA' is the binomial distribution with $(k-1)$ trials and probability $p_{A'|aA}$. The expectation is (k-1) $p_{A'|aA}$. Here $p_{A'|aA}$ is the probability of observing a wild-type A' next to an mutant a node in an Aa edge. Since we assume nodes with the same genotype are indistinguishable, we replace $p_{A'|aA}$ with $p_{A|aA}$.

The expected change under the second event (A replaces a) is

$$\begin{aligned}\sum_{k_{A'}} \frac{k_{a'} - k_{A'} - 1}{2Nk} P\Big(\Delta p_{Aa} = \frac{k_{a'} - k_{A'} - 1}{2Nk}\Big) &= \frac{1}{w} p_{Aa} \sum_{k_{A'}} \frac{k_{a'} - k_{A'} - 1}{2Nk} \text{Prob}(k_{A'}|Aa) \\ &= \frac{1}{w} p_{Aa} \sum_{k_{A'}} \frac{k - 2 - 2k_{A'}}{2Nk} \text{Prob}(k_{A'}|Aa). \\ &= \frac{1}{w}\frac{1}{2Nk} p_{Aa}(k - 2 - 2\mathbb{E}[k'_A|Aa]). \\ &= \frac{1}{w}\frac{1}{2Nk} p_{Aa}(k - 2 - 2(k-1)p_{A|Aa}).\end{aligned} \tag{11}$$

Writing w as a series expansion in s and ignoring higher order terms, for small s, the expected change in frequency of the Aa-type edges can be written as

$$\begin{aligned}\mathbb{E}[\Delta p_{Aa}] &= \frac{1}{2Nk} p_{Aa}\Big(2(k-1)(p_{A|aA} - p_{A|Aa}) - 2\Big) + \mathcal{O}(s) \\ &= \frac{1}{Nk}\Big((k-1)(p_{aAA} - p_{AaA}) - p_{Aa}\Big) + \mathcal{O}(s).\end{aligned} \tag{12}$$

From equation (12), we observe that the expected change in the frequency of p_{Aa} depends on the frequency of higher-order interactions, specifically the triples p_{AAa}, p_{AaA}. Here p_{AAa} is defined as the number of AAa of configurations divided by total number of triples in the graph. Similarly, the change in frequency over time of the triples depends on frequency of quartets. By induction, the change in frequency of $(n-1)$-tet depends on changes in frequencies of (n)-tet until n is equal to the population size N. This chain of dependencies can be simplified by the use of pair approximations, writing frequencies of (n)-tetas functions of frequencies of the lower levels of network organization.

Using approximations from [3], we write the frequencies of arbitrary triples of nodes XYZ as

$$p_{XYZ} = (1-\phi)p_{XY}p_{Z|Y} + \frac{N}{k}\phi p_{Y|X}p_{Z|Y}p_{X|Z}, \tag{13}$$

where ϕ is the transitivity or the number of closed triangles divided by the total number of triples.

We can rewrite equation (12) as

$$\mathbb{E}[\Delta p_{Aa}] = \frac{1}{k} p_{Aa}\left[(k-1)(1-\phi)\Big(1 - \frac{p_{Aa}}{p_A p_a}\Big) - 1\right] + \mathcal{O}(s). \tag{14}$$

We therefore need to keep track of frequency changes of the nodes and edges only.

We further observe that the expected change in node frequencies has terms of order s, while the expected

change in edge frequencies, in addition to terms of order s, also has terms independent of s. Therefore, in the limit of diminishing selection, the dynamics of the edges are faster than that of the nodes (terms independent of s dominate) and we can assume that the edge frequencies are at equilibrium in the timescale of the node dynamics. We write the equilibrium frequency of Aa edges as

$$p^*_{Aa} = (1-F)p_A p_a, \quad \text{where } F = \frac{1}{(k-1)(1-\phi)}. \tag{15}$$

$p^*_{Aa} = 0$ is also an equilibrium frequency, but this state is only reachable for non-zero p_a in disconnected graphs. Here, F is controlled by both the mean degree and the fraction of triangles of the network. Increasing the mean degree or decreasing the triangle count decreases F and increases the equilibrium number of Aa edges in the network.

We can now write the Kolmogorov backward equation for the combined node and edge dynamics and equation (15):

$$\frac{2+s}{2wN}(1-F)p_A p_a \frac{\partial^2 P}{\partial p_a^2} + \frac{s}{w}(1-F)p_A p_a \frac{\partial P}{\partial p_a} = \frac{\partial P}{\partial t}. \tag{16}$$

The probability of fixation given any initial mutant frequency is found by solving the KBE for zero

$$\frac{2+s}{2wN}\frac{\partial^2 P}{\partial p_a^2} + \frac{s}{w}\frac{\partial P}{\partial p_a} = 0, \tag{17}$$

which is identical to the KBE for the well-mixed model. This shows that, for regular graphs, the probability of fixation is unaltered by the higher-order topology.

The time to fixation conditioned on fixation of the mutant, on the other hand is, is not invariant to graph topology. We calculate the conditional fixation time from results on exit time in stochastic systems [4]. The time to fixation is given by solving the following equation for T:

$$\frac{2+s}{2wN}\frac{\partial^2}{\partial p_a^2}[T(p_a)P(p_a)] + \frac{s}{w}\frac{\partial}{\partial p_a}[T(p_a)P(p_a)] = -\frac{P(p_a)}{(1-F)p_A p_a}. \tag{18}$$

The solution of this equation is

$$T(p_a) = \frac{1}{1-F}T_{wm}(p_a), \tag{19}$$

where $T_{wm}(p_a)$ is the conditional fixation time for the well-mixed model. We obtain this value exactly using methods outlined in [5, 6].

Therefore, the time to fixation in regular graphs is a constant times the fixation time in the well-mixed

model, under weak selection. Our results show that increasing the triangle count increases the constant F and decreases the equilibrium number of Aa edges in the network, which translates into increased fixation time for the new mutant a.

2 Analytic approximation for graphs of heterogeneous degree

Here we present a full description of our analytic approach for arbitrary graphs of size N, under the Birth-death update rule. The main difference compared to the previous section is that we have to consider mutant frequencies in nodes of various degrees. Let $n_{i,a}$ be the number of mutants that occupy node of degree i and $n_{ij,Aa}$ be the number of edges that connects node A_i and a_j. We also define $p_{i,a}$ as $n_{i,a}$ divided by the total population size and $p_{ij,Aa}$ as $n_{ij,Aa}$ divided by the total number of edges $\sum n_{ij}$. We first analyze the quasi-equilibrium state of the deterministic neutral dynamics, and then describe the approximate stochastic dynamics using the quasi-equilibrium state variable.

The probability that a mutant occupying a node of degree j replaces a wild-type occupying a node of degree i is the probability of selecting a_j to reproduce and a neighboring A_i to die, which is given by

$$\begin{aligned} P(a_j \to A_i) &= \frac{1+s}{Nw} n_{j,a} \frac{n_{ji,aA}}{j n_{j,a}} \\ &= \frac{1+s}{Nw} n_{ji,aA} \frac{1}{j}. \end{aligned} \tag{20}$$

Here, w represents the mean fitness of the population. Using these update probabilities, we calculate the expected change of node types in one update as

$$\begin{aligned} a_j \to A_i: \quad \Delta n_{i,a} = +1 \quad \mathbb{E}[\Delta n_{i,a} | a_j \to A_i] &= +\frac{1+s}{Nwj} n_{ji,aA} \\ A_j \to a_i: \quad \Delta n_{i,a} = -1 \quad \mathbb{E}[\Delta n_{i,a} | A_j \to a_i] &= -\frac{1}{Nwj} n_{ji,Aa} \end{aligned} \tag{21}$$

This allows us to write out the total expected change in node type frequencies in one update

$$\mathbb{E}[\Delta n_{i,a}] = \frac{1}{Nw} \sum_j \frac{1}{j} \Big[(1+s) n_{ij,Aa} - n_{ij,aA} \Big]. \tag{22}$$

This leads to the following deterministic dynamics

$$\frac{d}{dt} n_{i,a} = \frac{1}{w} \sum_j \frac{1}{j} \Big[(1+s) n_{ij,Aa} - n_{ij,aA} \Big]. \tag{23}$$

The node dynamics therefore depends on the frequencies of edge types in the network. To get a full description of the deterministic dynamics, we also need to calculate the change in edge types during an update. For simplicity and clarity we only explicitly write out the terms independent of s, by assuming $Ns << 1$. The edge count $n_{ij,aA}$ can change in two types of events. The first type of event is a replacement event between a mutant occupying a node of degree i and wild-type occupying node of degree j. The expected update in $n_{ij,aA}$ is given by

$$\begin{aligned} a_i \to A_j: &\quad \Delta n_{ij,aA} = -1 \quad \mathbb{E}[\Delta n_{ij,aA}|a_i \to A_j] = -\frac{1}{Ni} n_{ij,aA} + o(s) \\ A_j \to a_i: &\quad \Delta n_{ij,aA} = -1 \quad \mathbb{E}[\Delta n_{ij,aA}|A_j \to a_i] = -\frac{1}{Nj} n_{ij,aA} + o(s). \end{aligned} \tag{24}$$

The combined expected change becomes

$$\mathbb{E}[\Delta n_{ij,aA}|i \leftrightarrow j] = -\frac{1}{N}\left(\frac{1}{i} + \frac{1}{j}\right) n_{ij,aA} + o(s). \tag{25}$$

The second type of event is when a third individual occupying a node of degree k replaces a node of degree i or j. There are four possible ways in which this can happen. For example, the probability of $a_k \to A_j a_i$ is

$$P(a_k \to A_j a_i) = \frac{1}{Nk} n_{kj,aA} \frac{n_{kji,aAa}}{n_{kj,aA}(j-1)} + o(s). \tag{26}$$

The first part of the first term is identical to equation (20) when s = 0. This is the probability that a_k is selected to replace A_j. The second part of the first term is the frequency of $a_k A_j a_i$ triplet in all triples containing the edge $a_k A_j$. In pair approximations, this frequency is the probability that an $a_k A_j$ edge forms an $a_k A_j a_i$ triplet. An $a_k A_j$ edge forms $j-1$ triplets centered around A_j. Out of these $j-1$ triplets, there are $j-1$ times the probability that an $a_k A_j$ edge forms an $a_k A_j a_i$ triplet on expectation, assuming the triplets are distributed binomially. We can write

$$\begin{aligned} a_k \to A_j a_i: &\quad \mathbb{E}[\Delta n_{ij,aA}|a_k \to A_j a_i] = -n_{kji,aAa}/(Nk) + o(s), \\ A_k \to a_j a_i: &\quad \mathbb{E}[\Delta n_{ij,aA}|A_k \to a_j a_i] = +n_{kji,Aaa}/(Nk) + o(s). \end{aligned} \tag{27}$$

The expected change for this scenario is given by

$$\mathbb{E}[\Delta n_{ij,aA}|k \to ji] = \sum_k \frac{1}{Nk}(n_{kji,Aaa} - n_{kji,aAa}) + o(s). \tag{28}$$

The other two possibilities are given by

$$
\begin{aligned}
a_k \to A_i A_j: &\quad \mathbb{E}[\Delta n_{ij,aA} | a_k \to A_i A_j] = +n_{kij,aAA}/(Nk) + o(s), \\
A_k \to a_i A_j: &\quad \mathbb{E}[\Delta n_{ij,aA} | A_k \to a_i A_j] = -n_{kij,AaA}/(Nk) + o(s).
\end{aligned}
\tag{29}
$$

The expected change in frequency can be written as

$$
\mathbb{E}[\Delta n_{ij,aA} | k \to ij] = \sum_k \frac{1}{Nk}(n_{kij,aAA} - n_{kij,AaA}) + o(s). \tag{30}
$$

By summing 25, 28, and 30, we write out the deterministic edge dynamics as

$$
\frac{d}{dt} n_{ij,aA} = -\left(\frac{1}{i} + \frac{1}{j}\right) n_{ij,aA} + \sum_k \frac{1}{k}(n_{kji,Aaa} - n_{kji,aAa} + n_{kij,aAA} - n_{kij,AaA}) + o(s). \tag{31}
$$

Since we assume $Ns \ll 1$, the quasi-equilibrium point is, therefore, calculated by solving the neutral dynamics for 0 (setting (23) and (31) to zero):

$$
\begin{cases}
0 = \sum_j \frac{1}{j}(n_{ij,Aa} - n_{ij,aA}) & \text{node equilibrium,} \\
0 = -\left(\frac{1}{i} + \frac{1}{j}\right) n_{ij,aA} + \sum_k \frac{1}{k}(n_{kji,Aaa} - n_{kji,aAa} + n_{kij,aAA} - n_{kij,AaA}) & \text{edge equilibrium.}
\end{cases}
\tag{32}
$$

Supplementary Figure B Panel A shows that the frequencies of edge types remain constant throughout most of the evolutionary trajectory. Therefore, an quasiequilibrium assumption gives an accurate approximation of the network dynamics.

We make two key approximations. Then first is a pair approximation, which is the generalization of the triple closure from the previous section. This simplifies the dynamics by expressing triple types as edge types and node types. We write

$$
n_{ijk,XYZ} = \frac{j-1}{j}\left((1-\phi)\frac{n_{ij,XY} n_{jk,YZ}}{n_{j,Y}} + \phi \frac{n\bar{d}}{ik} \frac{n_{ij,XY} n_{jk,YZ} n_{ik,XZ}}{n_{i,X} n_{j,Y} n_{k,Z}}\right), \tag{33}
$$

where $\bar{d}$ is the mean degree of the network. Information on the higher order description of network topology is lost under this assumption, but the number of equations that describes the network dynamics greatly

reduces. The second approximation assumes

$$\begin{cases} n_{ij,AA} = n_{ij} p_{A|i}(1 - c_{ij} p_{a|i}), \\ n_{ij,Aa} = n_{ij} c_{ij} p_{A|i} p_{a|j}, \\ n_{ij,aA} = n_{ij} c_{ij} p_{a|i} p_{A|j}, \\ n_{ij,aa} = n_{ij} p_{A|j}(1 - c_{ij} p_{a|i}). \end{cases} \tag{34}$$

Here, $p_{a|i} = p_{i,a}/p_i$ is the probability of finding an a node, conditioned on the node having i neighbors. The c_{ij} are quantities that capture the clustering of mutants due to network structure, leading to a decrease in the number of Aa edges, since they only exist on the boundaries of clusters.

Using approximations (33) and (34), we can simplify the equilibrium node frequencies and obtain

$$\begin{aligned} 0 &= \sum_j n_{ij} c_{ij} (p_{a|i} p_{A|j} - p_{A|i} p_{a|j}) \frac{1}{j} \\ &= \sum_j n_{ij} c_{ij} [p_{i|a}(1 - p_{a|j}) - (1 - p_{a|i}) p_{a|j}] \frac{1}{j} \\ &= \sum_j n_{ij} c_{ij} (p_{a|i} - p_{a|j}) \frac{1}{j}. \end{aligned} \tag{35}$$

This equation is satisfied if $p_{a|i} = p_{a|j}$, which means that the deterministic force will drive the mutant frequency to be uniform across all nodes. To write an expression for $p_{a|i}$, we introduce an intermediate variable q_a to connect the change in the deterministic neutral equilibrium to drift and selection. Specifically, we define q_a so that it is a martingale under the neutral dynamics

$$q_a = \frac{1}{\mathbb{E}[i^{-1}]} \sum_i p_i p_{a|i} \frac{1}{i}, \quad \text{where} \quad \mathbb{E}[i^{-1}] = \left(\sum_i p_i \frac{1}{i} \right)^{-1}. \tag{36}$$

Since $p_{a|i} = p_{a|j}$, observe that $q_a = p_{a|i} = p_{a|j}$ in the limit of large time and furthermore

$$\begin{aligned} \frac{d}{dt} q_a &= \frac{1}{\mathbb{E}[i^{-1}]} \frac{d}{dt} \sum_i p_{i,a} \frac{1}{i} \\ &= \frac{1}{\mathbb{E}[i^{-1}]} \sum_{ij} n_{ij} c_{ij} (p_{a|i} p_{A|j} - p_{A|i} p_{a|j}) \frac{1}{ij} \\ &= 0, \end{aligned} \tag{37}$$

which means that we can write $p_{a|i} = p_{a|j} = q_a(t = 0)$. This simplifies the approximation of edge dynamics to

only involve frequencies of mutants occupying nodes of various degrees and the total number of heterogeneous edges in the system. Equation (25) thus becomes

$$\mathbb{E}[\Delta n_{ij,aA}|i \leftrightarrow j] = -\left(\frac{1}{i}+\frac{1}{j}\right) c_{ij} n_{ij} q_a q_A. \tag{38}$$

Similarly, equation (28) can be rewritten as

$$\begin{aligned}
\mathbb{E}[\Delta n_{ij,aA}|k \to ji] &= \sum_k \frac{1}{k}(n_{kji,Aaa} - n_{kji,aAa}) \\
&= \frac{j-1}{j}\sum_k \frac{1}{k}\Bigg((1-\phi) n_{kj,Aa}\frac{n_{ji,aa}}{n_{j,a}} + \phi\frac{n\bar{d}}{ik}\frac{n_{kj,Aa}n_{ji,aa}n_{ik,aA}}{n_{k,A}n_{j,a}n_{i,a}} \\
&\qquad - (1-\phi)n_{kj,aA}\frac{n_{ji,Aa}}{n_{j,A}} - \phi\frac{n\bar{d}}{ik}\frac{n_{kj,aA}n_{ji,Aa}n_{ik,aa}}{n_{k,a}n_{j,A}n_{i,a}}\Bigg) \\
&= \frac{j-1}{j} q_A q_a \sum_k \frac{1}{k}\Bigg((1-\phi)n_{kj}c_{kj}\frac{n_{ji}(1-c_{ji}q_A)}{n_j} + \phi\frac{n\bar{d}}{ik}\frac{n_{kj}c_{kj}n_{ji}(1-c_{ji}q_A)n_{ik}c_{ik}}{n_k n_j n_i} \\
&\qquad - (1-\phi)n_{kj}c_{kj}\frac{n_{ji}c_{ji}q_a}{n_j} - \phi\frac{n\bar{d}}{ik}\frac{n_{kj}c_{kj}n_{ji}c_{ji}n_{ik}(1-c_{ik}q_A)}{n_k n_j n_i}\Bigg) \\
&= \frac{j-1}{j} q_A q_a \sum_k \frac{1}{k}\Bigg((1-\phi)\frac{n_{kj}n_{ji}}{n_j}c_{kj}(1-c_{ji}q_A) + \phi\frac{n\bar{d}}{ik}\frac{n_{kj}n_{ji}n_{ik}}{n_k n_j n_i}c_{kj}(1-c_{ji}q_A)c_{ik} \\
&\qquad - (1-\phi)\frac{n_{kj}n_{ji}}{n_j}c_{kj}c_{ji}q_a - \phi\frac{n\bar{d}}{ik}\frac{n_{kj}n_{ji}n_{ik}}{n_k n_j n_i}c_{kj}c_{ji}(1-c_{ik}q_A)\Bigg) \\
&= \frac{j-1}{j} q_A q_a \sum_k \frac{1}{k}\Bigg((1-\phi)\frac{n_{kj}n_{ji}}{n_j}c_{kj}(1-c_{ji}) + \phi\frac{n\bar{d}}{ik}\frac{n_{kj}n_{ji}n_{ik}}{n_k n_j n_i}c_{kj}(c_{ik}-c_{ji})\Bigg).
\end{aligned} \tag{39}$$

Equation (30) becomes

$$
\begin{aligned}
\mathbb{E}[\Delta n_{ij,aA}|k \to ij] &= \sum_k \frac{1}{k}(n_{kij,aAA} - n_{kij,AaA}) \\
&= \frac{i-1}{i} \sum_k \frac{1}{k} \Bigg((1-\phi) n_{ki,aA} \frac{n_{ij,AA}}{n_{i,A}} + \phi \frac{n\bar{d}}{jk} \frac{n_{ki,aA} n_{ij,AA} n_{jk,Aa}}{n_{k,a} n_{i,A} n_{j,A}} \\
&\qquad - (1-\phi) n_{ki,Aa} \frac{n_{ij,aA}}{n_{i,a}} - \phi \frac{n\bar{d}}{jk} \frac{n_{ki,Aa} n_{ij,aA} n_{jk,AA}}{n_{k,A} n_{i,a} n_{i,A}} \Bigg) \\
&= \frac{i-1}{i} q_A q_a \sum_k \frac{1}{k} \Bigg((1-\phi) n_{ki} c_{ki} \frac{n_{ij}(1 - c_{ij} q_a)}{n_i} + \phi \frac{n\bar{d}}{jk} \frac{n_{ki} c_{ki} n_{ij} (1 - c_{ij} q_a) n_{jk} c_{jk}}{n_k n_i n_j} \\
&\qquad - (1-\phi) n_{ki} c_{ki} \frac{n_{ij} c_{ij} q_A}{n_i} - \phi \frac{n\bar{d}}{jk} \frac{n_{ki} c_{ki} n_{ij} c_{ij} n_{jk} (1 - c_{jk} q_a)}{n_k n_i n_i} \Bigg) \\
&= \frac{i-1}{i} q_A q_a \sum_k \frac{1}{k} \Bigg((1-\phi) \frac{n_{ki} n_{ij}}{n_i} c_{ki}(1 - c_{ij} q_a) + \phi \frac{n\bar{d}}{jk} \frac{n_{ki} n_{ij} n_{jk}}{n_k n_i n_j} c_{ki}(1 - c_{ij} q_a) c_{jk} \\
&\qquad - (1-\phi) \frac{n_{ki} n_{ij}}{n_i} c_{ki} c_{ij} q_A - \phi \frac{n\bar{d}}{jk} \frac{n_{ki} n_{ij} n_{jk}}{n_k n_i n_i} c_{ki} c_{ij} (1 - c_{jk} q_a) \Bigg) \\
&= \frac{i-1}{i} q_A q_a \sum_k \frac{1}{k} \Bigg((1-\phi) \frac{n_{ki} n_{ij}}{n_i} c_{ki}(1 - c_{ij}) + \phi \frac{n\bar{d}}{jk} \frac{n_{ki} n_{ij} n_{jk}}{n_k n_i n_j} c_{ki} (c_{jk} - c_{ij}) \Bigg).
\end{aligned}
\tag{40}
$$

By summing equations (25), (28), and (30) we obtain the expected change in heterogeneous edge frequencies connecting i and j. Setting this to to zero we obtain

$$
\begin{aligned}
0 = &- \left(\frac{1}{i} + \frac{1}{j} \right) c_{ij} \\
&+ \frac{j-1}{j} \sum_k \frac{1}{k} \Bigg((1-\phi) \frac{n_{kj}}{n_j} c_{kj}(1 - c_{ji}) + \phi \frac{n\bar{d}}{ik} \frac{n_{kj} n_{ik}}{n_k n_j n_i} c_{kj} (c_{ik} - c_{ji}) \Bigg) \\
&+ \frac{i-1}{i} \sum_k \frac{1}{k} \Bigg((1-\phi) \frac{n_{ki}}{n_i} c_{ki}(1 - c_{ij}) + \phi \frac{n\bar{d}}{jk} \frac{n_{ki} n_{jk}}{n_k n_i n_j} c_{ki} (c_{jk} - c_{ij}) \Bigg).
\end{aligned}
\tag{41}
$$

Thus, we find c_{ij} by solving this quadratic system.

Analysis of the quasi-equilibrium stochastic dynamics

We can now analyze the stochastic dynamics by considering how the equilibrium frequency q_a changes when an individual on node of degree i is replaced by the opposite genotype occupying a node of degree j. The

two possible scenarios are

$$\begin{aligned} a_j \to A_i : \quad & \Delta q_a = \frac{1}{\mathbb{E}[i^{-1}]}\frac{1}{Ni} = +\Delta_i \\ A_j \to a_i : \quad & \Delta q_a = -\frac{1}{\mathbb{E}[i^{-1}]}\frac{1}{Ni} = -\Delta_i. \end{aligned} \tag{42}$$

To compute the total change in q_a when a mutant on node of degree i is replaced by the wildtype, we sum over the probabilities in (21), over j. This leads to

$$\begin{aligned} P\left(\Delta q_a = +\Delta_i\right) &= \frac{1+s}{Nw} q_a q_A \sum_j n_{ij} c_{ij} \frac{1}{j} \\ P\left(\Delta q_a = -\Delta_i\right) &= \frac{1}{Nw} q_a q_A \sum_j n_{ij} c_{ij} \frac{1}{j}. \end{aligned} \tag{43}$$

The first and second moment of the change in frequency of the mutant allele a, at every time step, can be written as:

$$\begin{aligned} \mathbb{E}[\Delta q_a] &= \frac{1}{Nw} s q_a q_A \sum_{ij} \Delta_i n_{ij} c_{ij} \frac{1}{j} = \frac{1}{N^2 w \mathbb{E}[i^{-1}]} s q_a q_A \sum_{ij} n_{ij} c_{ij} \frac{1}{ij} \\ \mathbb{E}[\Delta q_a^2] &= \frac{1}{Nw} (2+s) q_a q_A \sum_{ij} \Delta_i^2 n_{ij} c_{ij} \frac{1}{j} = \frac{1}{N^3 w \mathbb{E}[i^{-1}]^2} (2+s) q_a q_A \sum_{ij} n_{ij} c_{ij} \frac{1}{i^2 j}. \end{aligned} \tag{44}$$

Therefore, the per generation first and second moment of the change in frequency of the mutant allele a, at every time step, can be written as:

$$\begin{aligned} \mu[\Delta q_a] &= \frac{1}{Nw\mathbb{E}[i^{-1}]} s q_a q_A \sum_{ij} n_{ij} c_{ij} \frac{1}{ij} \\ \sigma[\Delta q_a] &\approx \frac{1}{N^2 w \mathbb{E}[i^{-1}]^2} (2+s) q_a q_A \sum_{ij} n_{ij} c_{ij} \frac{1}{i^2 j}, \end{aligned} \tag{45}$$

in the limit of weak selection. The diffusion approximation of the dynamics of q_a is given by

$$\frac{\partial P}{\partial t} = q_a q_A \left(\frac{2+s}{2Nw} \frac{\partial^2 P}{\partial q_a^2} \frac{1}{N\mathbb{E}[i^{-1}]^2} \sum_{ij} n_{ij} c_{ij} \frac{1}{i^2 j} + \frac{s}{w} \frac{\partial P}{\partial q_a} \frac{1}{N\mathbb{E}[i^{-1}]} \sum_{ij} n_{ij} c_{ij} \frac{1}{ij} \right) \tag{46}$$

We rewrite this as

$$\frac{\partial P}{\partial t} = q_a q_A \left(\frac{2+s}{2Nw} \lambda \frac{\partial^2 P}{\partial q_a^2} + \frac{s}{w} \lambda \alpha \frac{\partial P}{\partial q_a} \right), \tag{47}$$

where

$$\alpha = \mathbb{E}[i^{-1}] \sum_{ij} \frac{c_{ij} n_{ij}}{Nij} \left(\sum_{ij} \frac{c_{ij} n_{ij}}{Ni^2 j} \right)^{-1}, \tag{48}$$

is the amplification factor, and

$$\lambda = \mathbb{E}[i^{-1}]^{-2} \sum_{ij} \frac{c_{ij} n_{ij}}{N i^2 j} \tag{49}$$

is the acceleration factor. Solving for $P(q_a^0)$ gives the probability of fixation when starting with initial condition q_a^0.

To solve for the probability of fixation with one mutant appearing on any node, we find the corresponding q_a^0 and substitute into P. For mutants appearing uniformly in the graph, we find the probability of fixation, P_μ, by averaging $P(q_a^0)$ over all nodes.

Observe that

$$\begin{aligned} P_\mu &= \sum_i p_i P\left(\frac{1}{\mathbb{E}[i^{-1}]} \frac{1}{iN}\right) \\ &\approx \sum_i p_i \left[P(0) + \frac{1}{\mathbb{E}[i^{-1}]} \frac{1}{iN} P'(0)\right] \\ &= \sum_i p_i \frac{1}{\mathbb{E}[i^{-1}]} \frac{1}{iN} P'(0) \\ &\approx P\left(\frac{1}{\mathbb{E}[i^{-1}]} \sum_i p_i \frac{1}{iN}\right) \\ &= P\left(\frac{1}{N}\right). \end{aligned} \tag{50}$$

Hence, we can approximate the crossing time and probability for a mutant appearing on a random node of the network by solving for $P\left(\frac{1}{N}\right)$. The probability of fixation can be found by solving

$$q_a q_A \left(\frac{2+s}{Nw} \lambda \frac{\partial^2 P}{\partial q_a^2} + \frac{s}{w} \alpha \lambda \frac{\partial P}{\partial q_a}\right) = 0 \tag{51}$$

for $q_a = 1/N$ and weak selection s,

$$P\left(\frac{1}{N}\right) \approx \frac{1 - e^{-\alpha s}}{1 - e^{-\alpha N s}}. \tag{52}$$

The time to fixation can be found by solving

$$\frac{2+s}{2Nw} \frac{\partial^2}{\partial q_a^2} (T(q_a) P(q_a)) + \frac{\alpha s}{w} \frac{\partial}{\partial q_a} (T(q_a) P(q_a)) = -\frac{1}{\lambda} \frac{P(q_a)}{q_a q_A}. \tag{53}$$

The time to fixation is $1/\lambda$ times the fixation time for a well-mixed population with effective fitness $(1+\alpha s)$.

Therefore, for networks of heterogeneous degree, node and edge dynamics overall behave qualitatively similar to those of the k-regular graphs, with the dynamics of Aa edges faster than those of the nodes. The

frequencies of Aa edges equilibrate rapidly, as the mutant frequency increases in the population (**Supplementary Figure B Panel A**). These equilibrium Aa frequencies decrease as the triangle count increases (**Supplementary Figure B Panel B**). Unlike for regular graphs, the equilibrium Aa frequencies decrease by different amounts, with the strongest decreases observed in edges that connect low degree nodes. This difference is also affected by the mixing pattern of the network, with negligible difference in the reduction for graphs with low assortativity. This difference in magnitude causes the probability of fixation to change when the triangle count is altered. Intuitively, if all equilibrium edges are decreased by the same factor, they cancel in the Kolmogorov backward equation, resulting in no net change in the probability of fixation. This is no longer true if the reduction in equilibrium frequencies is not constant across all edge types. As a consequence, the change in probability and time to fixation depend on the equilibrium frequency of Aa edges that connect the lowest degree nodes in the graph (**Supplementary Figure B Panel C** and **Panel D**).

Our results generalize to commonly used network families. For example, let us consider small-world networks, with properties observed in many social networks [7]. The change in probability of fixation for both death-Birth and Birth-death process due to higher-order interactions is negligible for small and negative assortativity (**Supplementary Figure C Panel A**). As assortativity increases, the probability of fixation increases with increasing number of triangles in the network under the Birth-death process (**Supplementary Figure S3B**), and the probability of fixation decreases as triangle counts increase under the dearth-Birth process (**Supplementary Figure C Panel C**). Times to fixation increase as triangle counts increase for both processes (**Supplementary Figure C Panel B** and **Panel D**).

The next graph family we consider is generated using the Barabasi-Albert model of preferential attachment, which creates graphs with the scale-free property often found in social networks [8]. For this reason, they are typically used to study the spread of information or cultural norms [9]. The change in probability of fixation for both death-Birth and Birth-death processes is negligible for these graphs, while the time to fixation increases as triangle counts increase for both processes (**Supplementary Figure D**).

We also consider random geometric graphs which model spatially structured populations [10, 11]. In this family of graphs, nodes have spatial positions randomly drawn from a probability distribution to model spatially homogeneous populations (using the uniform distribution) or populations with heterogeneous spatial density (using the normal distribution). Once the spatial locations of the nodes are determined, the generating algorithm iterates through all pairs of nodes. An edge is created between two nodes if the pair-wise distance is below some prescribed threshold. The change in the probability of fixation for both death-Birth

and Birth-death processes due to higher-order interactions in the network is negligible, while the time to fixation increases as triangle counts increase for both processes (**Supplementary Figure E**).

3 Effects of 4-node Motifs on Evolutionary Dynamics

We also explore the effects of 4-node motifs on the evolutionary dynamics of the new mutation a. The dK-preserving edge-swap algorithm is only defined for $d = 3$. There is no edge-swap operation that is guaranteed to preserve the triplet distribution and the fraction of triangles in the network. However, in degree uniform graphs, edge swap does not affect the triplet distribution since all nodes have the same degree; only the ratio of open triples and triangles is altered. We tune the fraction of 4-node motifs (**Supplementary Figure F Panel A**), by randomly selecting edges for a 3K edge-swap and only accepting the change if the resulting graph contains the same number of triangles. We combine this with an optimization algorithm to get a range of graphs with different motif counts. The resulting algorithm can not, however, tune the occurrence of 4-motifs independent of other 4-motifs. The relationships between 4-cliques and other 4-motifs are shown in **Supplementary Figure F Panels B-F**.

Increasing the number of 4-cliques in the network decreases the time to fixation, while not influencing the probability of fixation in regular graphs (**Supplementary Figure G**). We only explicitly tune the number of 4-cliques since most other 4-motifs occurrence are correlated with the number of 4-cliques. **Supplementary Figure G** shows that the effects of the 4-clique on the time to fixation are negligible when the graph contains a low number of triangles. Motifs of order 4 only slightly begin to decrease the time to fixation when the number of triangles in the network is increased. It is surprising that the 3-cliques have the opposite effect on times to fixation compared to 4-cliques. However, the observed effects cannot be attributed to 4-cliques alone since increasing the number of 4-cliques can significantly reduce the number of semi-cliques and three-loop-outs, structurally similar to triangles, leading to a net negative influence on the time to fixation. These dependencies between the 4-motifs could explain the observed noise in the time to fixation. Future studies that modify four or higher node structures independently could further reveal their underlying roles in evolutionary dynamics. Results on higher-order interactions suggest that triangle counts are the most effective property in tuning temporal dynamics of a population since the dynamics induced by triangle counts are not affected by other 3-node structures.

References

[1] Hisashi Ohtsuki, Christoph Hauert, Erez Lieberman, and Martin A Nowak. A simple rule for the evolution of cooperation on graphs and social networks. *Nature*, 441(7092):502–505, 2006.

[2] Emanuele Pugliese and Claudio Castellano. Heterogeneous pair approximation for voter models on networks. *Europhysics Letters*, 88(5):58004, 2009.

[3] Thomas House and Matt J Keeling. Insights from unifying modern approximations to infections on networks. *Journal of The Royal Society Interface*, 8(54):67–73, 2011.

[4] Crispin Gardiner. *Stochastic methods*, volume 4. Springer Berlin, 2009.

[5] Warren John Ewens. *Mathematical population genetics: theoretical introduction*, volume 27. Springer, 2004.

[6] Tibor Antal and Istvan Scheuring. Fixation of strategies for an evolutionary game in finite populations. *Bulletin of Mathematical Biology*, 68(8):1923–1944, 2006.

[7] Duncan J Watts and Steven H Strogatz. Collective dynamics of small world networks. *Nature*, 393(6684):440–442, 1998.

[8] Albert-László Barabási and Réka Albert. Emergence of scaling in random networks. *Science*, 286(5439):509–512, 1999.

[9] Nicole Creanza, Oren Kolodny, and Marcus W Feldman. Cultural evolutionary theory: How culture evolves and why it matters. *Proceedings of the National Academy of Sciences*, 114(30):7782–7789, 2017.

[10] Bernard M Waxman. Routing of multipoint connections. *IEEE journal on selected areas in communications*, 6(9):1617–1622, 1988.

[11] Mathew Penrose et al. *Random geometric graphs*, volume 5. Oxford university press, 2003.

Supplementary Material: Supplementary Figures

List of Figures

A **Tuning the fraction of triangles in the network without keeping network mixing pattern constant can result in mistakenly assuming non-linear dependencies.** The starting graph is a preferential attachment graph with mean degree equal to five. The degree distribution is held constant. We vary the fraction of triangles in the graph, using edge swapping operations which also modify the mixing pattern of the network, as represented by the color legend. Here, $N = 100$ and $s = 0.05$. The dots represent ensemble averages across $5e6$ replicate Monte Carlo simulations. 20

B **Triangles decrease numbers of different types of Aa edges unequally in degree heterogeneous graphs.** The degree distribution is held constant as we vary the fraction of triangles in the graphs using edge swapping operations. Here $N = 100$ and $s = 0.05$. Color indicates the degree correlation of the network as a measure of mixing pattern. The dots represent ensemble averages across $5e6$ replicate Monte Carlo simulations. 21

C **Effects of the fraction of triangle in small-world graphs with various mixing patterns. Panels A** and **B** model the Birth-death process, while **Panels C** and **D** model the death-Birth process. We use small world graphs with mean degree equal to four. The dots represent ensemble averages across $5e6$ replicate Monte Carlo simulations. The degree distribution and graph assortativity are held constant, as we vary the fraction of triangles in the graphs. The fraction of triangles in the graph is tuned using edge swapping operations. Here $N = 100$ and $s = 0.05$. The colors indicate the assortativity of the network, as in the legend. 22

D **Effects of the fraction of triangle in preferential attachment graphs with various mixing patterns. Panels A** and **B** model the Birth-death process, while **Panels C** and **D** model the death-Birth process. We use preferential attachment graphs with mean degree equal to ten. The dots represent ensemble averages across $5e6$ replicate Monte Carlo simulations. The degree distribution and graph assortativity are held constant, as we vary the fraction of triangles in the graphs. The fraction of triangles in the graph is tuned using edge swapping operations. Here $N = 100$ and $s = 0.05$. The colors indicate the assortativity of the network, as in the legend. 23

E **Effects of the fraction of triangle in random geometric graphs with various mixing patterns. Panels A** and **B** model the Birth-death process, while **Panels C** and **D** model the death-Birth process. We use random geometric graphs with a cut-off radius of 0.2. The dots represent ensemble averages across $5e6$ replicate Monte Carlo simulations. The degree distribution and graph assortativity are held constant, as we vary the fraction of triangles in the graphs. The fraction of triangles in the graph is tuned using edge swapping operations. Here $N = 100$ and $s = 0.05$. The colors indicate the assortativity of the network, as in the legend. 24

F **Correlations of 4-motif counts in the graph.** We tune the count of 4-cliques in the graph using edge swaps and plot it against the resulting change in the counts of other higher order 4D structures in the graph. **Panel A** showcases the 4-order structures. **Panel B** shows the relationship between the 4-cliques and the stars. **Panel C** shows the relationship between 4-cliques and chains. **Panel D** shows the relationship between 4-cliques and three-loop-outs. **Panel E** shows the relationship between 4-cliques and boxes. **Panel F** shows the relationship between 4-cliques and semi-cliques. 25

G **Effects of the fraction of 4-node structures in the graph on evolutionary dynamics. Panel A**: 4-clique counts in the network do not affect the probability of fixation for regular graphs. **Panel B** Increasing 4-clique counts in the graph decreases fixation time. The dots represent ensemble averages across $5e6$ replicate Monte Carlo simulations. The colors represent the fraction of triangles in the graph. The graphs used are regular graphs with mean degree of 5 and size of $N = 100$. Here, selection strength $Ns = 5$. 26

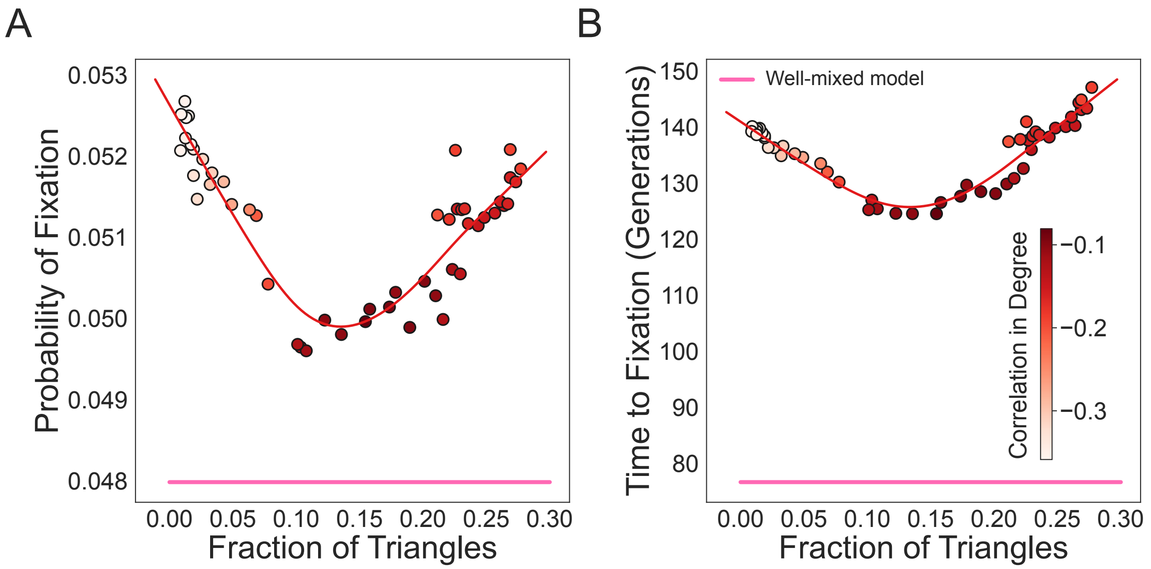

Supplementary Figure A: **Tuning the fraction of triangles in the network without keeping network mixing pattern constant can result in mistakenly assuming non-linear dependencies.** The starting graph is a preferential attachment graph with mean degree equal to five. The degree distribution is held constant. We vary the fraction of triangles in the graph, using edge swapping operations which also modify the mixing pattern of the network, as represented by the color legend. Here, $N = 100$ and $s = 0.05$. The dots represent ensemble averages across $5e6$ replicate Monte Carlo simulations.

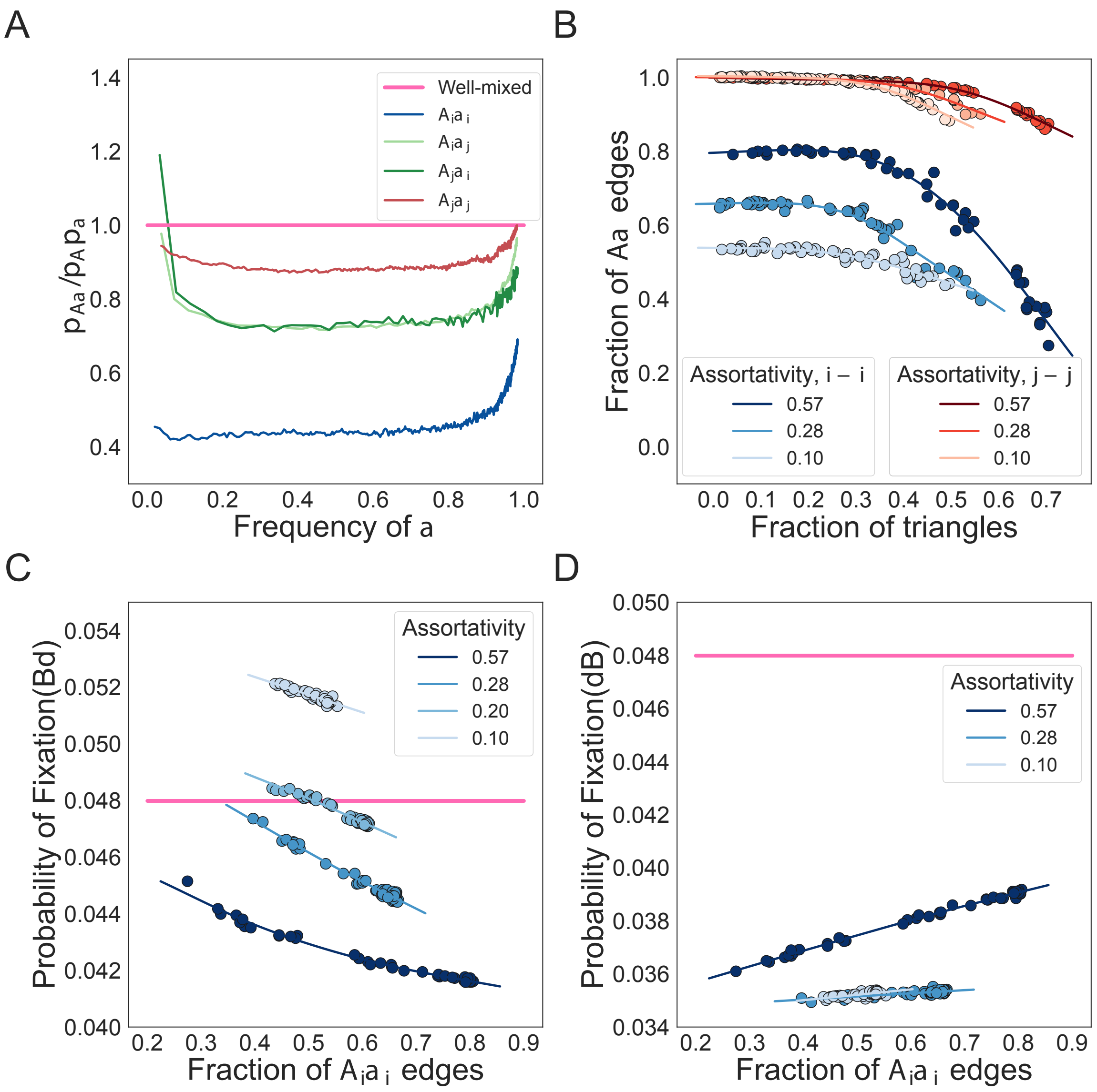

Supplementary Figure B: **Triangles decrease numbers of different types of** Aa **edges unequally in degree heterogeneous graphs.** The degree distribution is held constant as we vary the fraction of triangles in the graphs using edge swapping operations. Here $N = 100$ and $s = 0.05$. Color indicates the degree correlation of the network as a measure of mixing pattern. The dots represent ensemble averages across $5e6$ replicate Monte Carlo simulations.

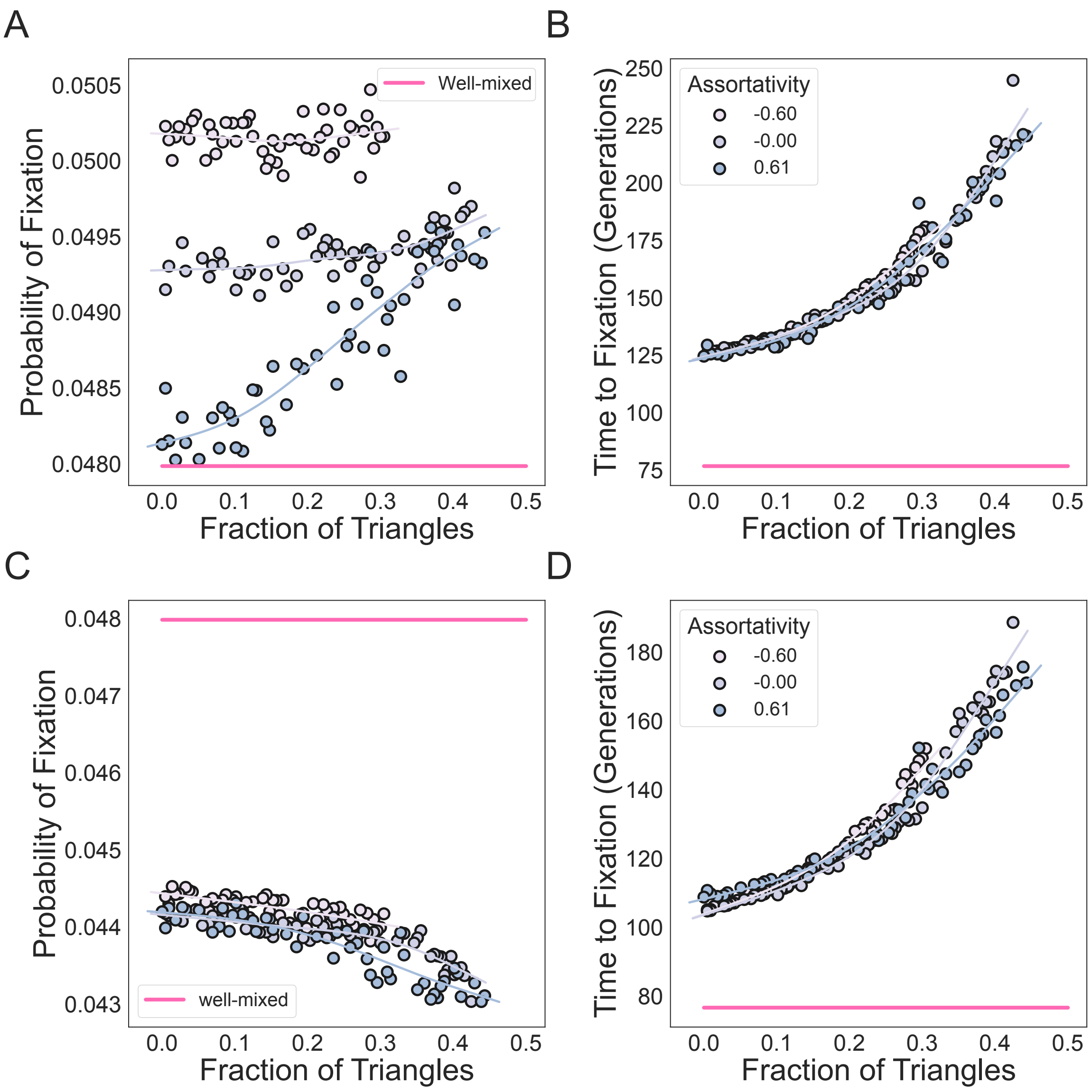

Supplementary Figure C: **Effects of the fraction of triangle in small-world graphs with various mixing patterns. Panels A** and **B** model the Birth-death process, while **Panels C** and **D** model the death-Birth process. We use small world graphs with mean degree equal to four. The dots represent ensemble averages across $5e6$ replicate Monte Carlo simulations. The degree distribution and graph assortativity are held constant, as we vary the fraction of triangles in the graphs. The fraction of triangles in the graph is tuned using edge swapping operations. Here $N = 100$ and $s = 0.05$. The colors indicate the assortativity of the network, as in the legend.

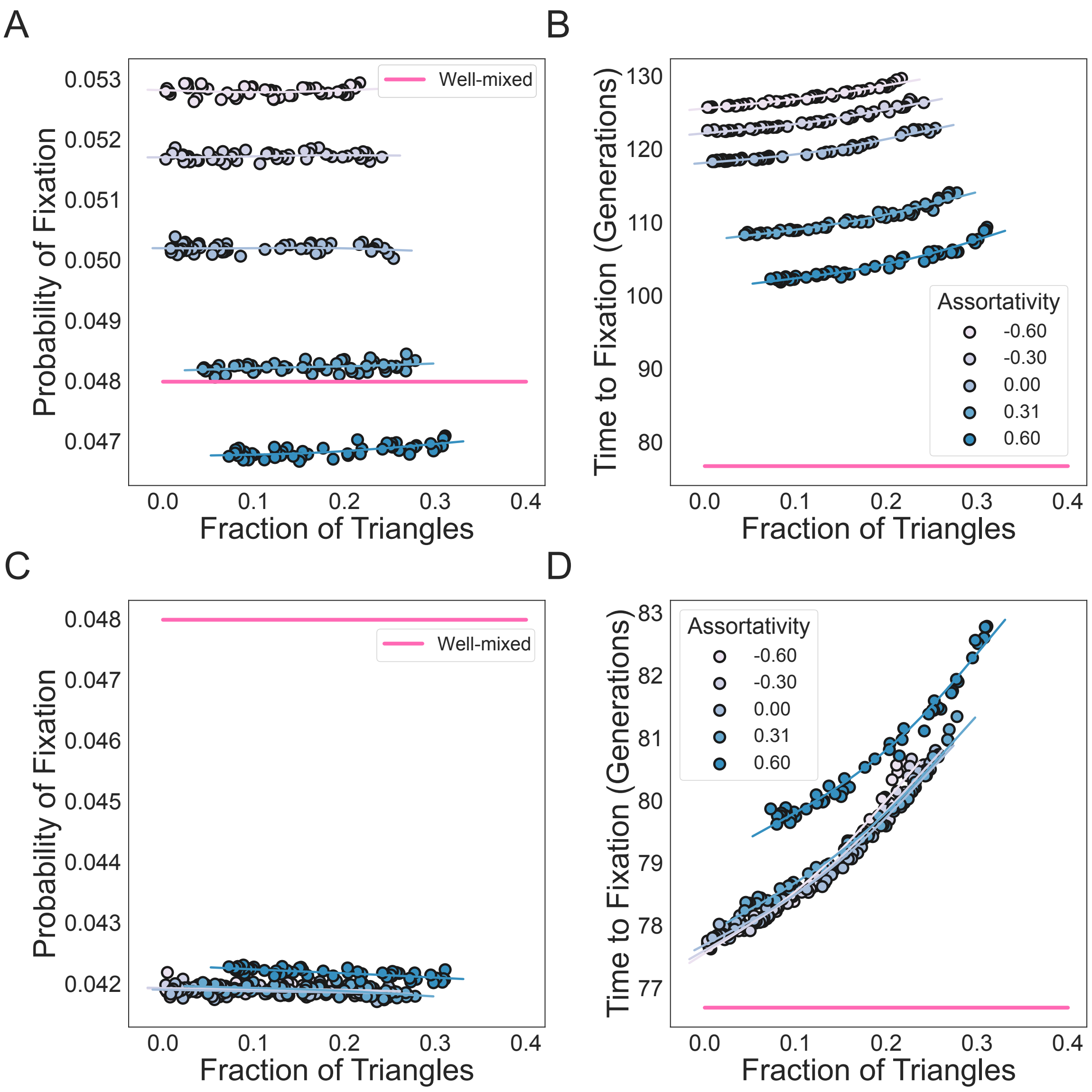

Supplementary Figure D: **Effects of the fraction of triangle in preferential attachment graphs with various mixing patterns. Panels A** and **B** model the Birth-death process, while **Panels C** and **D** model the death-Birth process. We use preferential attachment graphs with mean degree equal to ten. The dots represent ensemble averages across 5e6 replicate Monte Carlo simulations. The degree distribution and graph assortativity are held constant, as we vary the fraction of triangles in the graphs. The fraction of triangles in the graph is tuned using edge swapping operations. Here $N = 100$ and $s = 0.05$. The colors indicate the assortativity of the network, as in the legend.

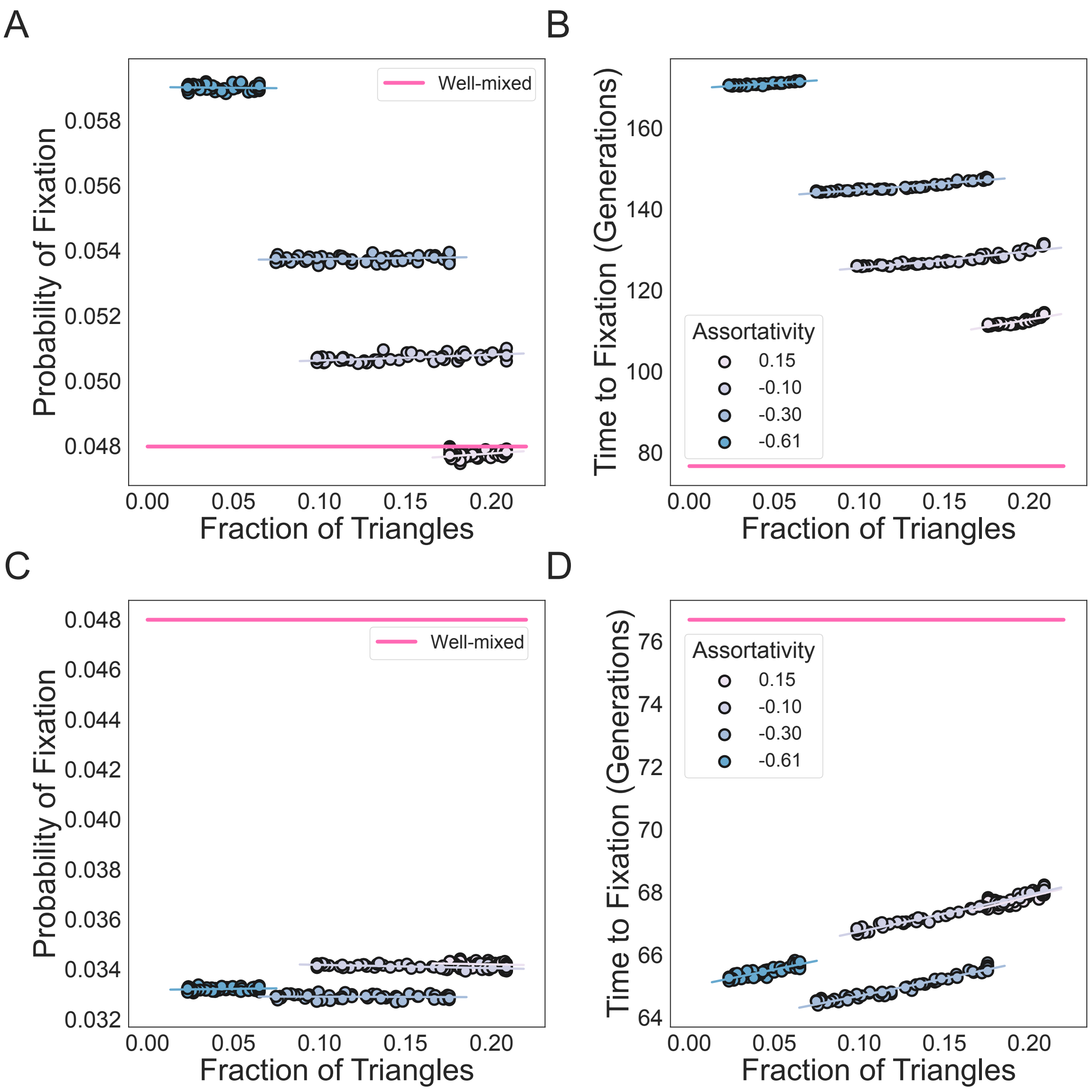

Supplementary Figure E: **Effects of the fraction of triangle in random geometric graphs with various mixing patterns. Panels A** and **B** model the Birth-death process, while **Panels C** and **D** model the death-Birth process. We use random geometric graphs with a cut-off radius of 0.2. The dots represent ensemble averages across 5e6 replicate Monte Carlo simulations. The degree distribution and graph assortativity are held constant, as we vary the fraction of triangles in the graphs. The fraction of triangles in the graph is tuned using edge swapping operations. Here $N = 100$ and $s = 0.05$. The colors indicate the assortativity of the network, as in the legend.

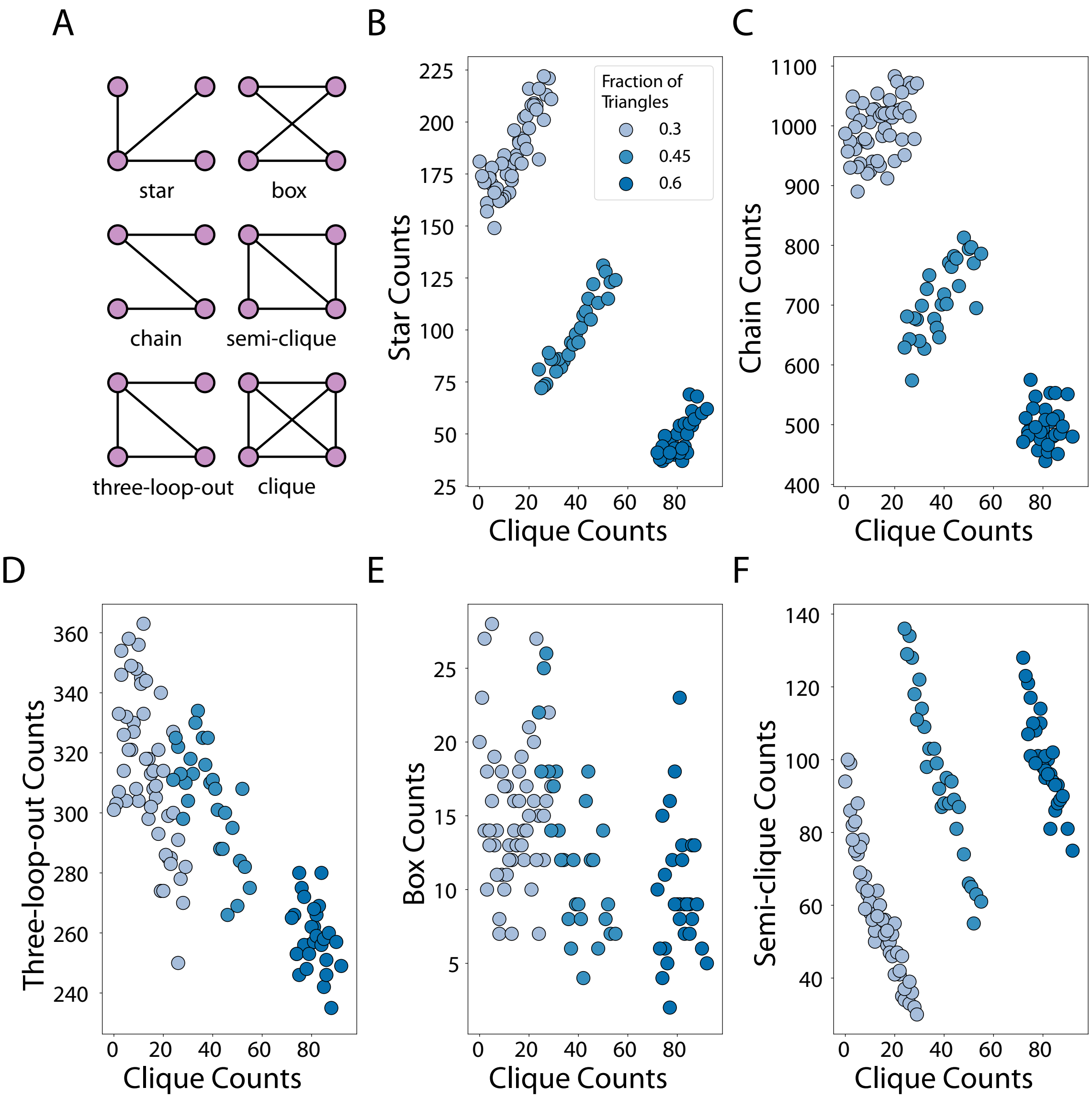

Supplementary Figure F: **Correlations of 4-motif counts in the graph.** We tune the count of 4-cliques in the graph using edge swaps and plot it against the resulting change in the counts of other higher order 4D structures in the graph. **Panel A** showcases the 4-order structures. **Panel B** shows the relationship between the 4-cliques and the stars. **Panel C** shows the relationship between 4-cliques and chains. **Panel D** shows the relationship between 4-cliques and three-loop-outs. **Panel E** shows the relationship between 4-cliques and boxes. **Panel F** shows the relationship between 4-cliques and semi-cliques.

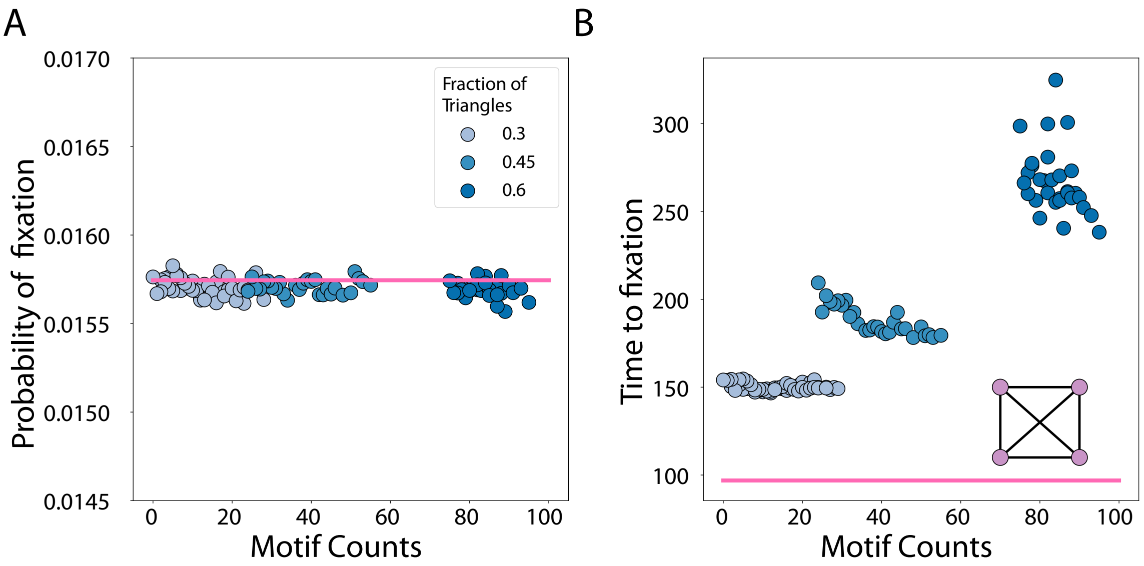

Supplementary Figure G: **Effects of the fraction of 4-node structures in the graph on evolutionary dynamics.** **Panel A**: 4-clique counts in the network do not affect the probability of fixation for regular graphs. **Panel B** Increasing 4-clique counts in the graph decreases fixation time. The dots represent ensemble averages across $5e6$ replicate Monte Carlo simulations. The colors represent the fraction of triangles in the graph. The graphs used are regular graphs with mean degree of 5 and size of $N = 100$. Here, selection strength $Ns = 5$.
